# Study of Selected Physical-Mechanical Properties of Corn Grains Important from the Point of View of Mechanical Processing Systems Designing

**DOI:** 10.3390/ma14061467

**Published:** 2021-03-17

**Authors:** Weronika Kruszelnicka

**Affiliations:** Department of Machines and Technical Systems, Faculty of Mechanical Engineering, University of Science and Technology in Bydgoszcz, 85-796 Bydgoszcz, Poland; weronika.kruszelnicka@utp.edu.pl

**Keywords:** corn, grinding, compression test, rupture energy, stiffness, biomass, breakage probability

## Abstract

Mechanical properties of corn grains are of key importance in a design of processing machines whose energy demand depends on these properties. The aim of this study is to determine the selected mechanical properties of corn grains and the rupture energy. The research problem was formulated as questions: (1) How much force and energy is needed to induce a rupture of corn grain maintaining good quality of the product of processing (mixing, grinding transport)? (2) Can empirical distributions of the studied physical-mechanical properties be described by means of probability distributions provided by the literature? (3) Is there a relationship between the corn grain size and the selected mechanical properties, as well as rupture energy? In order to achieve the goals, the selected physical properties (size, volume) of corn grains have been distinguished and a static compression test has been carried out on an Instron 5966 testing machine. The results indicate a significant scatter of the results in terms of size, grain shape, forces, energy, and deformation corresponding to the point of inflection, bioyiled point, and rupture point. It has also been indicated that empirical distributions of the analyzed properties can be described by means of distributions known from the literature, e.g., gamma, Weibull or lognormal distributions. It has been confirmed that mechanical properties such as force, energy, and stress that cause rupture depend on the grain size, more precisely, the grain thickness—there are negative relations between thickness and force, energy and stress in relation to the point of inflection, bioyiled point, and rupture point.

## 1. Introduction

The processing of biological materials is characterized by specific conditions caused, among others, by properties of the processed biological material including: Hardness, compression and shear strength, moisture, bulk density, compressibility, agglomeration ability, and adhesive properties [1]. The biological diversity of plant materials, even within one species, makes modeling of processing machines and devices in terms of efficiency and energy consumption more difficult [2,3,4,5]. It implies the need to explore properties of materials in terms of their processability. Determination of the relations between the processed material, the machine structural components, and the processes applied is an important issue from the point of view of ecology. Knowledge of the relations can be used for improvement of the process and quality of the end product, reduction in energy consumption, and the amount of waste, as well as its rational disposal according to the rules of sustainable development [1,6,7,8,9].

Grains of cultivated plants, e.g., cereals, *Oryza sativa* (rice), *Glycine max* (soya), *Zea mays* (corn), *Pisum sativum* (pease), *Linum usitatissimum* (flax), *Brassica napus* (rape), etc. are used in processing. Grains are usually used for consumption and livestock feeding, though recently they are increasingly used for energy purposes, e.g., rape for production of bio-diesel and corn grains for production of boiler fuel [10]. The use of production waste for energy purposes has become a common practice, e.g., oil extrusion waste can be a precious substrate for biogas plants and pellets are produced from rice hulls [10]. Grains can be used in catalysis processes.

Among the above listed grains, this is the corn that makes up the largest cultivation area, and subsequently is the most commonly processed grain material. Corn kernels play a significant role in the agri-food industry [11,12]. The mechanical properties of cobs, stems, and corn kernels determine the construction and operational parameters of the equipment intended for cutting, harvesting, and processing (e.g., grinding) of this plant [13,14,15,16,17,18]. They also affect the power consumption and energy consumption of cutting and grinding machines and equipment [19,20,21,22,23,24]. Since reduction in energy intensity is one of the key rules of sustainable economy [25,26,27,28], determining the forces needed to break grains is of key importance when developing the grinding process energy and environmental efficiency indicators, as well as modeling grinding and crushing processes with the use of the discrete element method DEM [15,29]. Determining the mechanical properties of biomaterial grains, including corn, requires the use of specialized research equipment with high measuring accuracy, which is primarily associated with the internal structure of granular materials of plant origin, that is, significantly different from the internal structure of metals [30,31].

Current research results indicate the variability of grain strength properties, depending on the species, internal structure, glassiness or moisture content [32]. Strength properties in turn, affect the conditions of work (energy) and power of a machine to be used for grain comminution [33]. It has been proven that more energy is needed for comminution of hard biological materials than for the soft ones [34,35,36], as in the case of materials with higher moisture and glassiness—both an increase in moisture and glassiness causes an increase in force and energy demand in the process of grain comminution [3,37,38,39,40,41]. A relationship between the grinding energy and the grain mass (energy increase along with mass increase) was observed for grains of cereals, e.g., wheat [2] geometric features of grains (thickness) and the force and work of crushing [4].

The previous research on the mechanical properties of corn has been focused, among others, on determination of the cutting forces and energy of cobs for different harvesting dates, and it has been shown that subsequent corn harvest is associated with lower cutting forces and lower energy demand [30]. Relationships between hardness and the internal structure of corn kernels have also been investigated [42]. In [43], in turn, the authors have examined, among others, the relationship between the corn grain size and moisture. They showed that size and humidity are not related to grain hardness. In [44], physical properties of corn kernels depending on humidity were studied, and it was shown that the size, sphericity, and density of corn kernels increases with their moisture content. Similar conclusions are presented in research [45,46,47] and [48], where additionally the models of rupture energy regression and destructive force depending on humidity have been determined. Other physical and mechanical properties determined for corn grains include the angle of repose and coefficients of friction [11,29,45,47,49,50]. Not many works deal with the estimation of mechanical properties of grains, and the available ones differ in the scope of testing methodology, primarily, devices and conditions for carrying out strength tests. The mechanical properties and energy of corn grinding depending on moisture are presented in works [31,51,52,53]. Soyoye et al. [54] studied the physical-mechanical properties of corn depending on the grain orientation in a testing machine. Zhang et al. [55] studied the impact of shear speed of corn stalks and cobs on strength properties, shear force, and energy. The impact of grain drying on their hardness and susceptibility to comminution has also been assessed in [56]. Works [31,47,57] ambiguously describe how the crack energy was calculated (or measured). It was not precisely defined which moment of rupture they relate to. The literature provides attempts to describe materials crack probability by means of known distributions. They refer, however, rather to hard materials, mostly to minerals and rocks [58,59,60,61,62]. There is a shortage of this type of studies for biological materials such as corn grains.

The aim of this study is the determination of selected physical-mechanical properties of corn grains and their rupture energy, which need to be known in the design of machines and manufacturing processes of the analyzed biomaterials and identification of the relationships between mechanical properties and the grain size. The research problem was formulated in the form of questions: (1) What force and energy is needed to induce a rupture of corn grain while maintaining an appropriate quality of the product during processing (mixing, grinding, transport)? (2) Can empirical distributions of the investigated physical-mechanical properties be described by means of probability distributions? (3) Is there a relationship between the corn grain size and the selected mechanical properties, as well as rupture energy? In order to provide answers to the above questions, an experiment was carried out for 100 corn grains, which allowed to determine parameters of the particle shape and size, values of forces, energy, and stresses characteristic for the point of inflection (a point, in which inclination of the force-deformation curve starts decreasing), bioyield point (point corresponding to the yield point during compression), and rupture point (the point on the force-deformation corresponding to the force that induces rupture) using a compression test. The results were subject to a statistical analysis, and probability distributions were determined for the analyzed values.

The remaining part of the paper includes Section 2 which contains a description of the preparation and the methods used to determine the size and shape of corn grain parameters, as well as the methods used to identify the mechanical properties and perform a statistical analysis. Section 3 is devoted to an analysis of the results, and the last section presents the most important conclusions.

## 2. Materials and Methods

### 2.1. Sample Preparation

Grains of the commonly grown in Poland corn (variety Amaizi CS, Caussade, Strzelin, Poland) were used in the tests. A corn kernel is made of 82% of endosperm, about 12% is an embryo, and the remaining part consists of other elements, i.e., the root part and fruit-seed cover [63,64]. Grains were separated from cobs and subjected to initial cleaning (they were purchased in this form, packed in a 50 kg bag). A representative general sample for tests, weighing 1 kg, was collected in accordance with PN-EN ISO 24333: 2012P [65]. Then, in accordance with the PN-EN ISO 24333: 2012P [65] standard, laboratory samples (weighing 125 g) were divided using the PT100 sample divider (Retsh, GmbH, Haan, Germany). Grains were subjected to conditioning, prior to tests they were kept in a climatic chamber for KBK-65W for 48 h (Wamed, Warszawa, Poland) with forced air circulation in a temperature of 20 °C to stabilize the moisture of the samples. After conditioning, the moisture was determined by means of the weight method with the use of moisture analyzer MAC 210/NP (RADWAG, Radom, Poland). The method involves determining the percentage mass losses during drying grains in a temperature of 105 °C [66]. The moisture is determined based on the difference in the sample mass before and after drying, according to Formula [67]:

*W_m_* = ((*m*_1_ − *m*_2_)/*m*_1_)·100% (1)

where *W_m_* is the total moisture of the sample, %, *m*_1_ is the mass of the sample before drying, g, and *m*_2_ is the mass of the sample after drying, g.

Thus, the moisture was 12.68 ± 0.01%. From one of 125 g of the laboratory sample, 100 representative grains with no sign of damage and no cracks were selected for the tests. Prior to the experiment, the samples were kept in a refrigerator in a temperature of 8 °C, in ziplock bags. The samples were left in the bags for 16 h before the experiment, in the room where the experiment was conducted to heat the samples to ambient temperature (21 °C).

### 2.2. Research Methods

**Figure 1:**
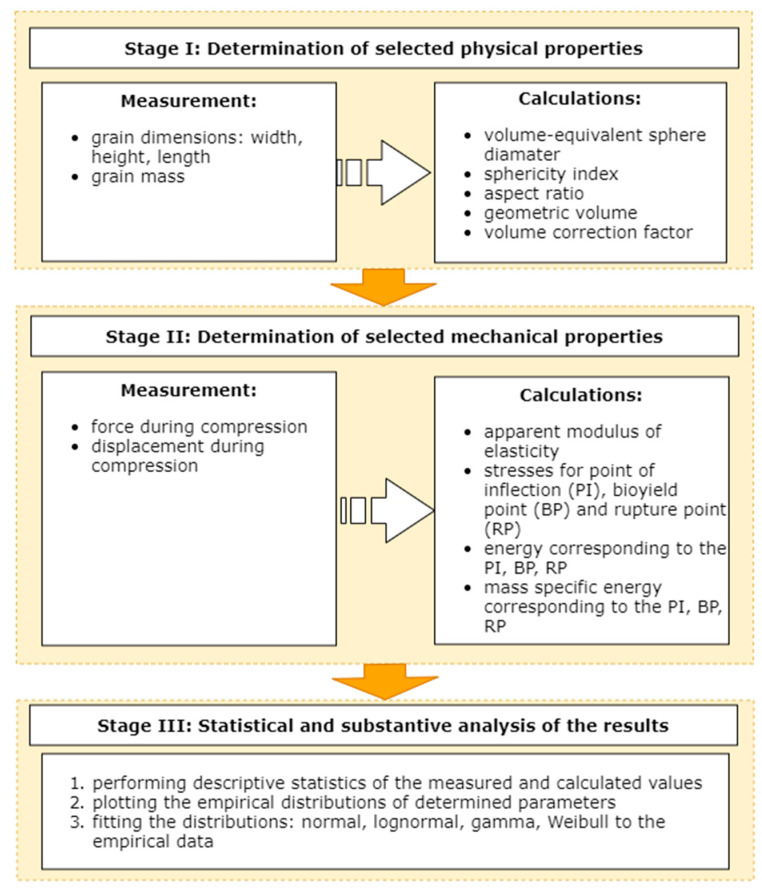
The flowchart of the study design.

The research on physical-mechanical properties of corn grains was divided into three parts: (a) Measurement of physical characteristics involving measurement of the grain dimensions, (b) compression test and determination of mechanical properties and the rupture energy, (c) analysis of the results. Figure 1 presents the flowchart of the study design.

#### 2.2.1. The Measurement of the Physical Properties

Length *a*_1_, width *a*_2_, and height *a*_3_ were measured for each grain with the electrical vernier caliper with a level of accuracy equal to ±0.01 mm. Based on the obtained values of the grain particular dimensions, the following quantities were calculated:

- Volume-equivalent sphere diameter *D_E_*, that is, diameter of a sphere of the same volume *V_T_* as the tested grain [68]:
  (2) $$D_{E}={\left(\frac{6}{\pi }V_{T}\right)}^{1/3}$$
- Sphericity index *f*, which defines the ratio of the grain volume to the volume described on the grain sphere with a diameter equal to the grain length *a*_1_ [69]:
  *f* = (*a*_1_·*a*_2_·*a*_3_)^1/3^/*a*_1_ (3)
- Aspect ratio *R*_a_, which expresses the ratio of width (*a*_2_) to the grain length (*a*_1_) [64]:
  *R_a_* = *a*_2_/*a*_1_ (4)
- Geometric volume *V_g_*, which corresponds to the volume of an ellipsoid with dimensions *a*_1_, *a*_2_, and *a*_3_ [64]:
  *_Vg_* = π·*a*_1_·*a*_2_·*a*_3_/6 (5)

Grain weight *m* was determined using an analytical scale AS 220/C/2 (RADWAG, Radom, Poland) with an accuracy level equal to ±0.001 g. Based on the values of grain mass *m* and its true density *ρ_T_* = 1.2 g·cm^−3^ [70,71], true volume *V_T_* [44,72] was calculated:

*V_T_* = *m*/*ρ_T_* (6)

During the experiment, the correction factor *k_v_* was determined, which allowed estimating the true grain volume (that resulting from mass and density *ρ_T_*) based on the knowledge of grain dimensions. This coefficient can be determined from [72]:

*k_V_* = *V_g_*/*V_T_* (7)

#### 2.2.2. Determination of Mechanical Properties

**Figure 2:**
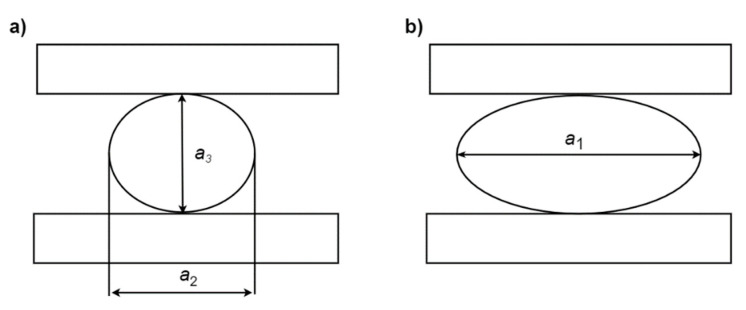
The corn grain position in the Instron 5966 testing machine. (**a**) Front view, (**b**) side view.

A static compression test was carried out on an Instron 5966 universal testing machine (Instron, Norwood, MA, USA) with the load speed vs. equal to 30 mm·min^−1^. The sample was placed between two parallel surfaces (the strength machine table and a head) in the way shown in Figure 2, according to ASAE S368.4 [73] standard. The ASAE S368.4 [73] was created especially for determining the mechanical properties of food materials of convex shape, such as fruits and vegetables, seeds, and grains. The corn grains are qualified to the group of materials for which this standard could be used. The corn grains are characterized by the convex and irregular shape, which will affect the course of the force-deformation curve, and this standard explains how to calculate the Young’s modulus and strength taking into account precisely the irregular shape of the grain. The ASAE S368.4 [73] provides the step-by-step description on how to calculate the semi-minor and semi-major axes of the contact area for different loading geometries and in consequence, the compression area for grain specimen, its strength, and Young’s modulus.

**Figure 3:**
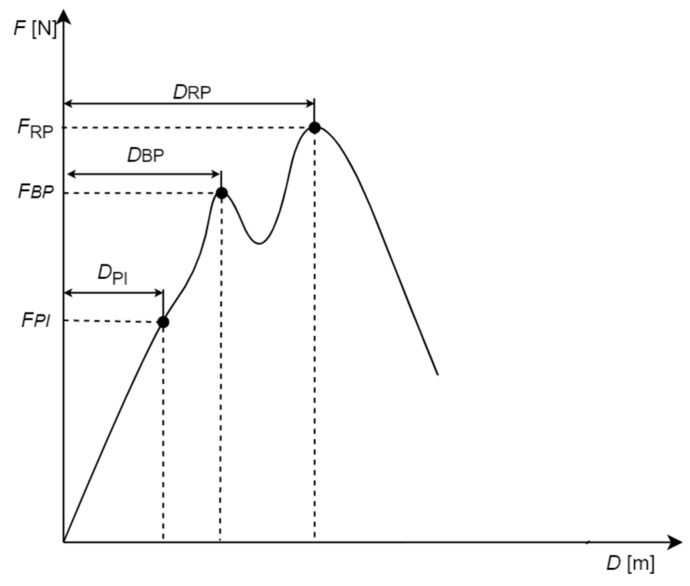
Characteristic points determined on the force-deformation curve obtained from the corn grain compression test.

Based on force-deformation curves values of forces *F_PI_*, *F_BP_*, and *F_RP_* were determined corresponding to the point of inflection (PI), bioyield point (BP), and rupture point (RP) and corresponding to them deformation *D_PI_*, *D_BP_*, and *D_RP_*, as shown in Figure 3.

According to the ASAE S368.4 [73] standard, the point of inflection is a point, in which inclination of the force-deformation curve starts decreasing and the bioyield point corresponds to the yield point, whereas RP indicates the value of the force (stress) that induces cracking. Forces and deformations for RP and BP were read directly from the diagrams of the force-deformation curve, whereas forces and deformations for PI were determined through an approximation of the force deformation curve by means of the appropriate functions, and the next second derivative of the approximated curves was calculated to be compared to zero in order to determine the points suspected of being points of inflexion. Next, the condition of the second derivative sign change in the zero place was checked. The inflexion point was accepted to be such a value of force and deformation, in which the second derivative of the approximation function was equal to zero which was a place where the function changed the sign from positive to negative.

The apparent modulus of elasticity was determined based on a dependence for the case of compression of nonsymmetric, convex grains between two parallel plates [73]:

(8) $$E=\frac{0.338F\left(1-\mu^{2}\right)}{D^{3/2}}{\left[K_{U}{\left(\frac{1}{R_{U}}+\frac{1}{R_{U}^{\prime }}\right)}^{1/3}+K_{L}{\left(\frac{1}{R_{L}}+\frac{1}{R_{L}^{\prime }}\right)}^{1/3}\right]}^{3/2}$$

where *E* is the apparent modulus of elasticity, Pa, *D* is the strain, m, *µ* is the Poisson coefficient, *F* is the strain causing force, N, *R_U_* is the minimal grain curve radius in the point of contact with the upper horizontal plate, *R_U_*′ is the maximal grain curve radius in the point of contact with the upper horizontal plate, *R_L_* is the minimal grain curve radius in the point of contact with the lower horizontal plate, *R_L_*′ is the maximal grain curve radius in the point of contact with the lower horizontal plate, and *K_U_*, *K_L_* are the constants resulting from the curve of grain being in contact with the plate.

*µ =* 0.2 [74] was accepted for corn. Constants *K_U_* and *K_L_* depend on the cosine of *θ* angle. In the ASAE S368.4 standard [73], there are tabular values of *K* for given values of *cosθ*. The following dependencies are determined for grains of complicated shape which are in contact with a flat plate *cosθ* [73]:

(9) $$\cos\theta =\frac{\frac{1}{R_{U(L)}}-\frac{1}{R_{U(L)}^{\prime }}}{\frac{1}{R_{U(L)}}+\frac{1}{R_{U(L)}^{\prime }}}$$

When the values of *cosθ* ranged between the values presented in [73], interpolation was used to determine the value of *K*.

The radii of the grain surface curve were determined by a computing method presented in [73]. It was assumed that the maximal and minimal radii of the contact curve for the upper and lower plate are the same. The minimal radius of the contact curve was calculated based on Dependence (10), whereas the maximal radius was calculated based on Dependence (11):

*R*_*U*(*L*)_ = *a*_3_/2 (10)

(11) $$R\prime_{U\left(L\right)}=\frac{a_{3}^{2}+\frac{a_{1}^{2}}{4}}{2a_{3}}$$

In the next step, the maximal stresses that occur in the sample under the impact of compression force were determined for three points PI, BP, and RP, according to Dependence [73]:

(12) $$S_{\max}=\frac{1.5F_{x}}{\pi ab}$$

where *F* is the deformation causing force, N, *a* is the semi-major axis, m, and *b* is the semi-minor axis, m.

(13) $$a=c_{1}{\left[\frac{3F\left(\kappa_{g}+\kappa_{p}\right)}{2}{\left(\frac{1}{R_{U\left(L\right)}}+\frac{1}{R_{U(L)}^{\prime }}\right)}^{-1}\right]}^{1/3}$$

(14) $$b=c_{2}{\left[\frac{3F\left(\kappa_{g}+\kappa_{p}\right)}{2}{\left(\frac{1}{R_{U\left(L\right)}}+\frac{1}{R_{U(L)}^{\prime }}\right)}^{-1}\right]}^{1/3}$$

where *c*_1_, *c*_2_ are the constants determined on the basis of knowledge of *cosθ*, *κ_g_* is the grain material constant, and *κ_p_* is the plate material constant.

(15) $$\kappa_{g(p)}=\frac{1-\mu_{g(p)}^{2}}{E_{g(p)}}$$

#### 2.2.3. Point of Inflection, Bioyield Point, and Rupture (Fracture) Energy

The energy needed for grain destruction was determined on the basis of Equation (16) [75,76]:

(16) $$E_{\left(PI,\;BP,\;RP\right)}=\int_{D_{1}}^{D_{\left(PI,\;BP,\;RP\right)}}FdD$$

where *E*_(*PI*, *BP*, *RP*)_ is the energy input until occurrence of PI, BP, respectively and RP, J, *F* is the force, N, and *dD* is the deformation corresponding PI, BP, RP, mm, respectively.

The energy (work) values during compression of one grain is the area under graph *F* = *f*(*D*) (Figure 3). Energy values *E_PI_* were calculated for deformation *D_PI_* caused by force *F_PI_*; *E_BP_* were calculated for *D_BP_* deformation caused by *F_BP_* force; and *E_RP_* were calculated for deformation *D_RP_* caused by force *F_RP_*.

According to Tavares et al. [58], the mass specific energy corresponding to points *E_m_*_(*PI, BP, RP*)_ was determined based on Equation (17):

(17) $$E_{m(PI,BP,RP)}=\frac{1}{m}\int_{D_{1}}^{D_{\left(PI,BP,RP\right)}}FdD=\frac{E_{\left(PI,BP,RP\right)}}{m}$$

where *m* is the mass of a single grain expressed in kilograms.

### 2.3. Analytical Methods

The statistical analysis of the results was conducted in Origin Pro 2020. In this study, descriptive statistics of measured values of physical-mechanical properties and the rupture were determined. The univariate analysis was used for calculating the distribution of a single variable, including its central tendency (average and median) as well as dispersion (the range of the data-set), and measures of spread (standard deviation). Normality of distributions was assessed by means of the normality test of Shapiro-Wilk, which is one of the most commonly used and strongest normality tests. For distributions, for which the normal one was rejected by the Shapiro-Wilk test, fitting of density functions other than the normal observed in lognormal, Weibull, and Gamma (Table 1) distribution tests, was assessed by the Kolmogorov-Smirnov goodness-of-fit test accepting a significance level of 0.05. Both the Shapiro-Wilk test and modified Kolmogorov-Smirnov goodness-of-fit test verify the zero hypothesis that a given sample comes from a population with a tested distribution. If a test reaches significance *p* < 0.05 the zero hypothesis is rejected, if *p* > 0.05 it is assumed that the data come from a population with a tested distribution. The final choice of the result distribution model was made on the basis of probability with the use of a comparison to find out which distribution points arrange along the reference line.

A significant scatter of results of force, strength, and deformation energy is observed both for the energy for biomaterials and brittle materials [59,72,76]. Therefore, it is necessary to make the right choice of a property variability distribution so as to be able to describe the occurring phenomena as accurately as possible. In works [59,76], a method of order statistics was used, by means of which empirical function of a given property occurrence probability can be determined in the form of probability distributions. In order to determine cumulated probability distributions of force, strength, and mass specific energy, their values were structured in an ascending order ranking *i* = 1,2,…,*N*, for particular observations. Cumulated empirical probability distribution of the analyzed property can then be determined from a dependence resulting from the Hanzen score method [59,76]:

(22) $$P\left(X_{i}\right)=\frac{i-0.5}{N}$$

where *P(X_i_*) is the value of cumulated distribution of probability of a given property occurrence, *X_i_* defines the analyzed property, here the value of force, strength, and mass specific energy, and *N* is the number of observations. In this way, cumulated distributions of a given property occurrence probability can be determined on the basis of the results of experimental tests. As known from the literature, probability distribution functions can be matched to the obtained distributions. Similar to [59], the fitting of known probability distributions to the experiment data was analyzed. Three distributions most commonly used for crack probability description were tested: Cumulated lognormal distribution, cumulated Weibull distribution, and cumulated gamma distribution (Table 2) [58,59,62,76]. In order to match the distributions, the Levenberg-Marquardt nonlinear optimization algorithm was used. The choice of the best fitted distribution was made on the basis of the function ranking according to the value of determination coefficient R^2^.

The earlier tests of dependencies between the shape, size, and mass of the particles [4,59,77,78] imply that the size of particles has an impact on the value of forces, stresses, and energy of grinding. In order to verify the dependence between particle size, strength, and grinding energy, the analyzed particles were divided into four groups according to the grain thickness *a*_3_: 1—(4.0–4.5) mm, 2—(4.5–5.0) mm, 3—(5.0–5.5) mm, 4—(>5.5) mm. The grain thickness was chosen to be the diversifying value since earlier tests showed crack energy changes for this size [4]. Cumulated probability distributions of the analyzed properties and their medians (*X*_50_—*X* denotes the analyzed property), were determined for a given group. Then, the Pearson analysis of correlation was performed to study the relationship between the grain size and median values of forces, strength, and energy. The significance level *p* < 0.05 was adopted.

## 3. Results and Discussion

### 3.1. Physical Properties of Corn Grains

**Figure 4:**
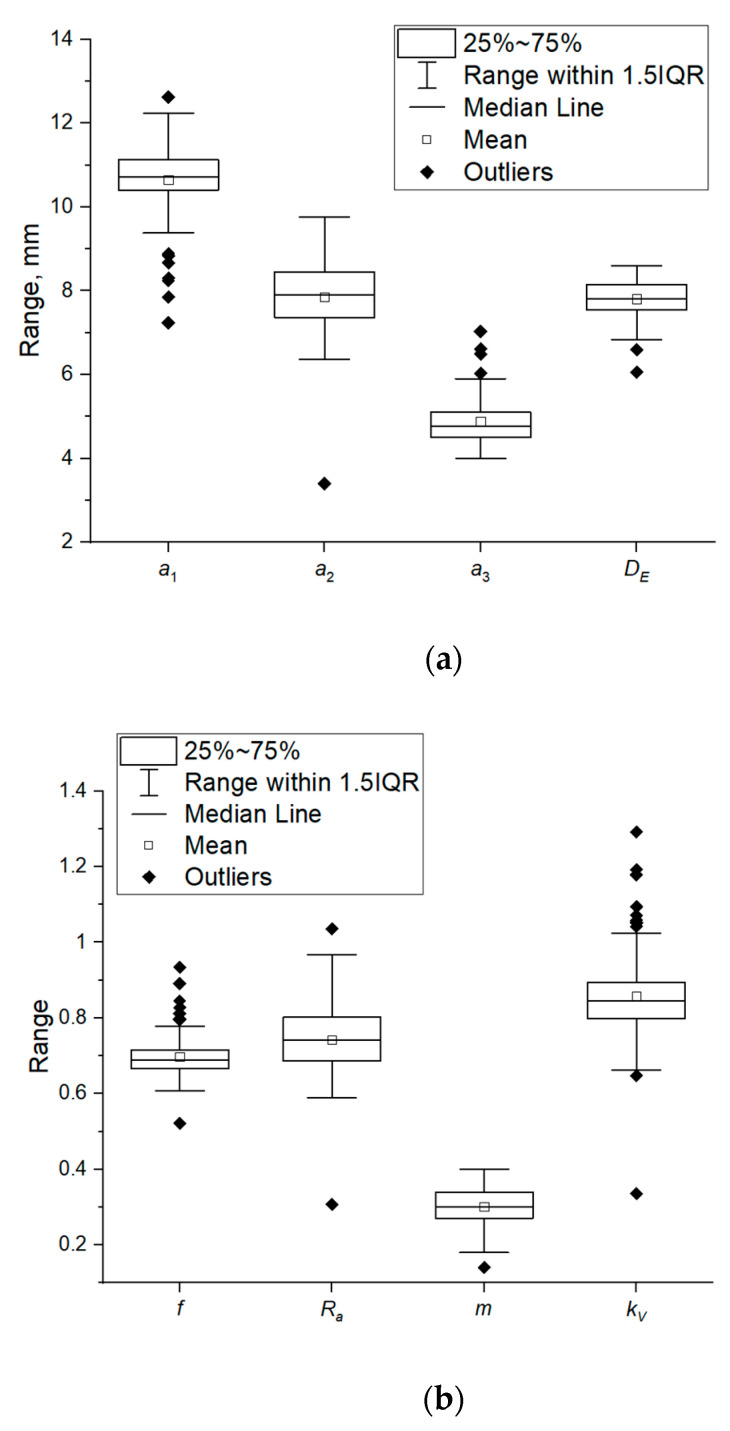

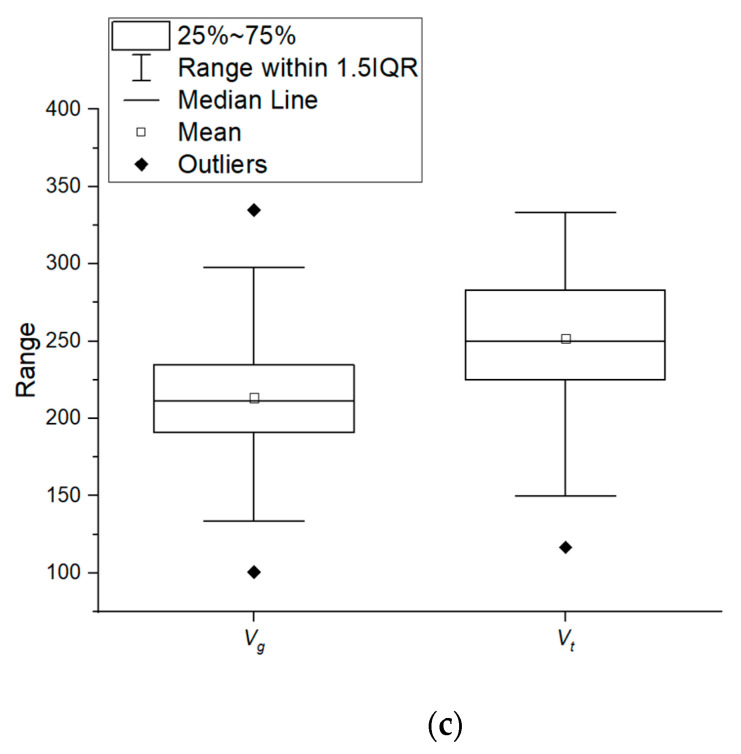
Box charts of values of the selected physical properties of corn grains, (**a**) properties connected with size: length (*a_1_*), volume-equivalent sphere diameter (*D_E_*), width (*a*_2_), and height (*a*_3_), (**b**) properties connected with shape and volume: aspect ratio (*R_a_*), sphercity index (*f*), mass (*m*), volume correction factor (*k_V_*), (**c**) grains volume: true volume (*V_t_*), geometric volume (*V_g_*).

The first stage of the study involved determination of the grain physical properties: Length *a*_1_, width *a*_2_, height *a*_3_, volume equivalent sphere diameter, aspect ratio, sphericity index, mass, geometric volume, true volume, and volume correction factor *k_v_*. Figure 4 presents the results of statistical analysis of the tested physical properties. The average length of corn grains was 10.65 mm, the average width was 7.85 mm, and the average height was 4.88 mm (Table 3).

**Figure A1:**
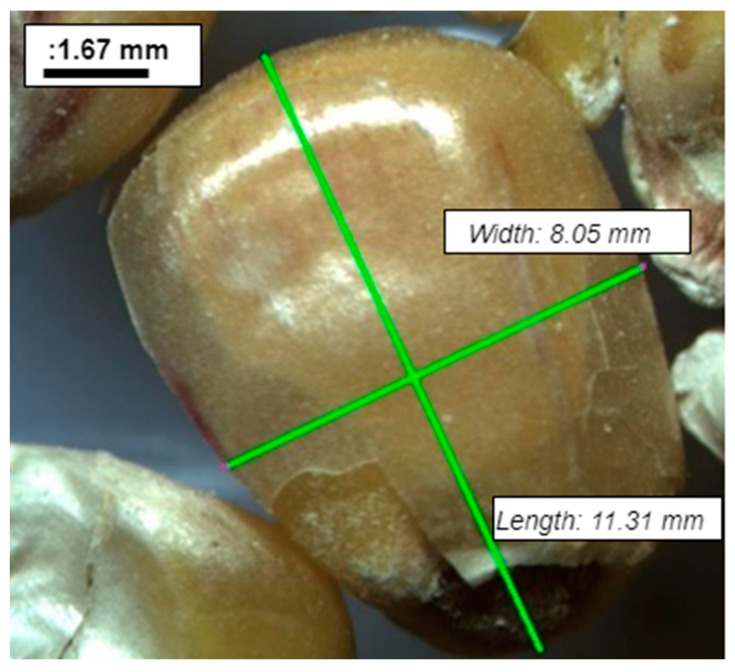
Sample photo showing the shape and size of corn grain of the studied variety. The photo was taken at a 15 times magnification.

Figure A1 shows an exemplary photo of corn grain of the studied variety with the marked dimensions. The tested corn grains are characterized by medium sphericity (which is also indicated by the sphericity index values (see Table 3 and Figure 4)), rounded edges, and shiny hull surface (Figure A1). The average true volume was equal to 251.67 mm^3^, while the average geometric volume calculated on the basis of measured dimensions was 213.40 mm^3^. The correction volume coefficient *k_v_* calculated from Equation (6) takes the average value equal to 0.858.

The results of corn grain dimensions *a*_1_, *a*_2_, and *a*_3_, are similar, although slightly smaller than those reported in the literature by other researchers, e.g., [46] and [43], for Large-IMIC, Medium-IMIC, and Large-Puma types, as well slightly higher for Small-Puma and Medium-Puma types (IMIC and Puma are the hybrid varieties of corn grains, the adjectives—large, medium, small—denote the diversification of a variety in terms of grain size). Differences in dimensions may be caused primarily by the difference in the varieties and types of the studied corn grains, the country of origin, grain humidity, and growing conditions of grains.

**Figure 5:**
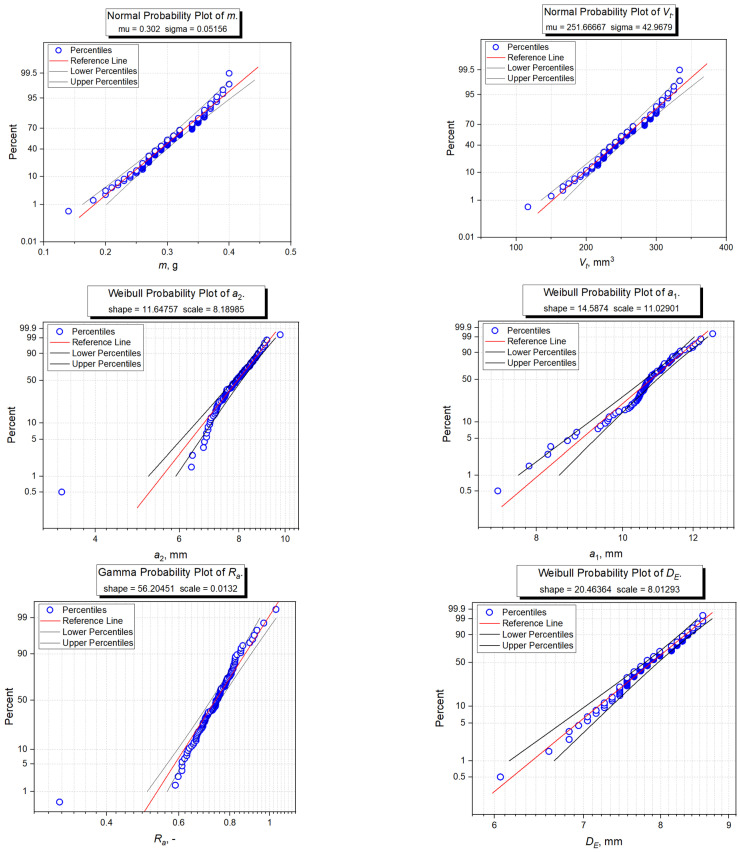

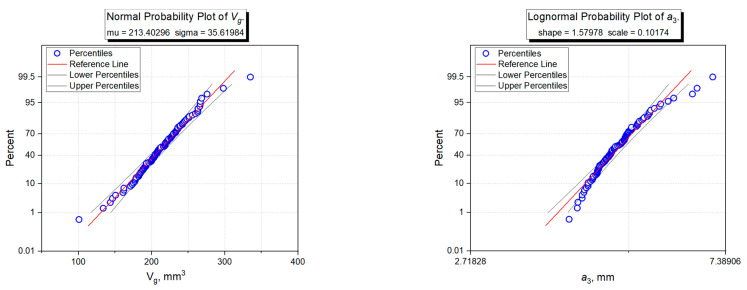
Probability plots of tested distributions fitted to empirical data of the selected physical properties of corn grain such as mass (*m*), true volume (*V_t_*), geometric volume (*V_g_*), length (*a_1_*), aspect ratio (*R_a_*), volume-equivalent sphere diameter (*D_E_*), width (*a*_2_), and height (*a*_3_). The blue point corresponds to the percentiles of empirical data. The red curve is the reference line for the tested distribution. The more empirical data are placed along the reference line, the better the theoretical distribution fits the experimental data.

Empirical data of probability distributions were fitted based on descriptive statistics, Shapiro-Wilk normality test, and the modified Kolmogorov-Smirnov distribution test. A distribution of *m* mass values, geometric volume *V_g_*, and true volume *V_T_* was accepted to be normal, according to the Shapiro-Wilk test (Table 4). The probability plots and the distribution parameters are presented in Figure 5. For the remaining properties of grains, the *p*-value was lower than the adopted significance level (*p* < 0.05) in the Shapiro-Wilk test, which suggested a rejection of the hypothesis that the tested samples come from a population with a normal distribution. The results of Kolmogorov-Smirnov tests indicated that height *a*_3_ of grains is consistent with the lognormal distribution and length *a*_1_ with the Weibull distribution (Table 4, Figure 5). The results for width *a*_2_ and aspect ratio *R_a_* indicated that the values of the examined parameters could come from Weibull or Gamma distributions (Table 4). Based on the probability plots (Figure 5) it was found that the distribution of results for width *a*_2_ is better described by Weibull distribution, whereas the aspect ratio is better described by gamma distribution. For volume-equivalent sphere diameter *D_E_*, the distribution of data is best described by Weibull distribution (Figure 5), though based on the Kolmogorov-Smirnov test (Table 4), it was found that the data can come from both the population with lognormal distribution and gamma distribution. When comparing, however, probability plots, Weibull distribution was found to be the best fitted. In the case of sphericity index *f* and volume coefficient *k_V_* none of the analyzed distributions was not fitted to the data.

While analyzing the results attention must be focused on a significant scatter of particle shape and size results. Thus, the earlier described in the literature [32,34,35,36] diversity of plant materials within one species and variety was confirmed.

Corn is one of the most commonly processed materials due to its wide application in food, fodder, chemical, cosmetic, and even power industries. A variety of machines and devices are used for processing, starting with machines for harvesting, cleaning, classification, and grinding. The results regarding both the size and shape, as well as probability distribution of the analyzed properties are of utilitarian character and can be used for optimization of structural features of machines and devices designed for corn processing such as: Mixers, conveyors, sieve screens, devices for cleaning, and classifying grains in terms of size and mass which can contribute to an improvement in efficiency of these devices, as well as their energy consumption which is of key importance in terms of sustainable development.

The knowledge of the particle size, in particular, the size distributions is of key significance for a design, operation, and maintenance of machines for corn grain harvesting [79,80]. Properly matched sieve hole sizes of the working units of harvesters can contribute to grain loss reduction during the harvest. In the case of screens and classifiers, it is possible to increase the accuracy of grain classification and their cleaning, thus raising the product quality. Knowing the particle size distributions is not without importance for the grinding processes and basically for the design and operation of grinders such as: Crushers, mills, roller mills, and disc mills. The size of grains determines, among others, the choice of structural features of working spaces, e.g., the size of the inter-roller gaps of roller grinders and crushers, as well as the working gap of multi-disc grinders. Knowing the size of particles can be useful in a design of these machines, taking into consideration, among others, the possibility of the gap regulation to be adjusted to a given class of grain size.

The presented results for grain size and shape are also indispensable elements of computer simulation models of mixing, transporting, and comminution processes based on the discrete element method (DEM) [37]. Recently, DEM is a method which has been increasingly used in the simulation of machine operation and optimization, devices, and processing of loose material. The precise representation of the particle shape and size affects the accuracy of a DEM model and prediction of grain behavior motion [81,82,83].

### 3.2. Strength Properties and Fracture Energy

**Figure 6:**
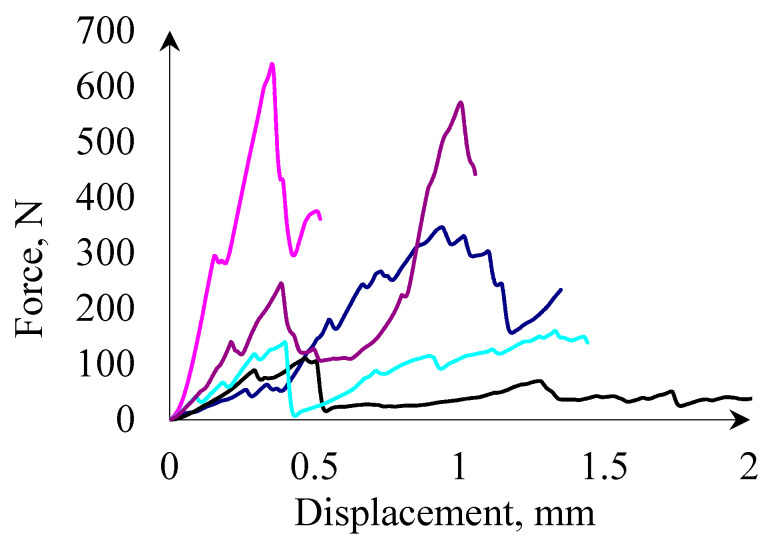
Example results of the compression test for five corn grains from 100 tested.

**Figure 7:**
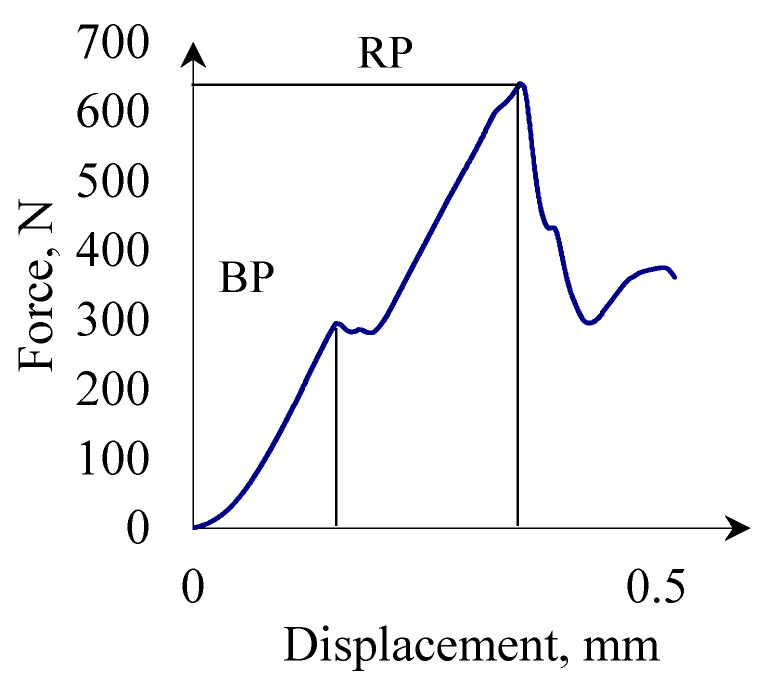
The force-deformation curve representing the cracking process for corn grains.

Figure 6 and Figure 7 present typical force displacement curves for a corn grain compression test. Based on the presented curves, it can be stated that the crack proceeds differently for each grain. This is caused by differences in the internal structure of each grain, which is characteristic for biomaterials. Changes in the internal structure cause changes in the hardness of the grains, therefore for harder grains, applying the same force will cause less deformation than in the case of less hard grains. Differences in the shape of the force-displacement curves are also caused by changes in the contact surface during grain compression, as well as differences in the shape of the grains themselves. As the research on the shape and size of the grains has shown, each grain was characterized by a different size, in addition, the curvature for each grain is also different, therefore, during compression for the same displacements, a different contact surface will occur. For more convex grains in the initial phase, the contact area of the grain and the pressing surface may be brought to a point and the contact area will increase with the compression, in such a situation the displacements may be greater with lower forces than, for example, for flatter grains, when already in the initial stage of compression the contact area is larger and at the same time the bigger part of the grain volume is compressed. Noticeable are the characteristic points in the force-displacement graph marked as *BP* and *RP* (Figure 7). The point marked as *BP* symbolizes the grain elasticity limit, while the *RP* corresponds to the forces causing the grain fracture into smaller fragments. Similar conclusions are presented for other biomaterials, for example, rice [38], wheat [84,85] grains, etc. [86]. The presented crack propagations of brittle materials, e.g., are provided in [59]. Occurrence of an area of plastic deformation characteristic of ductile materials is noticeable.

**Figure 8:**
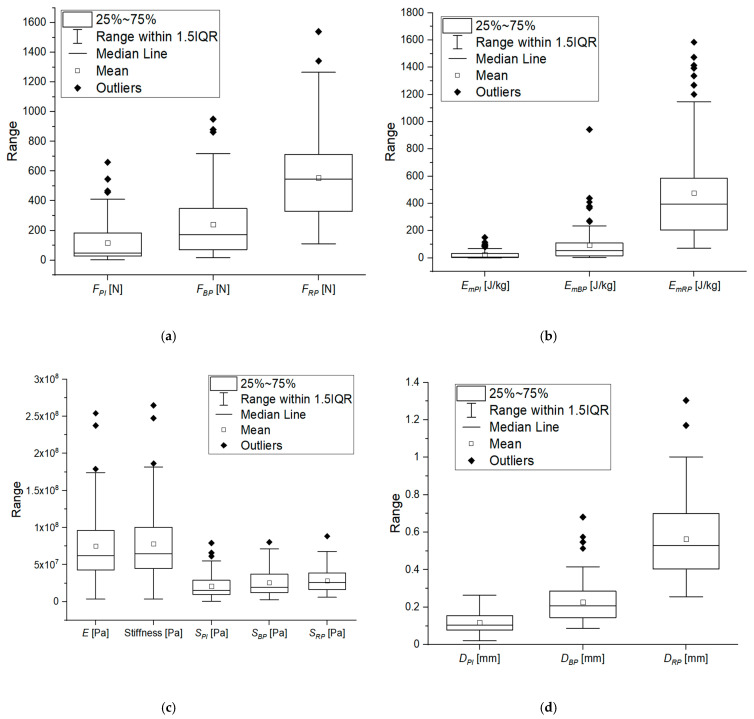
Box charts of strength parameters for corn grains determined in a compression test, (**a**) forces: *F_PI_* corresponding to point of inflection, *F_BP_* corresponding to bioyield point, *F_RP_* causing breakage, (**b**) specific energy: *E_mPI_* corresponding to point of inflection, *E_mBP_* corresponding to bioyield point, *E_mRP_* needed to break the grain, (**c**) young modulus *E*, stiffness and stresses: *S_PI_* corresponding to point of infection, *S_BP_* corresponding to bioyield point, *S_RP_* corresponding to grain breakage, (**d**) deformation: *D_PI_* corresponding to point of inflection, *D_BP_* corresponding to bioyield point, *D_RP_* corresponding to grain breakage.

The scopes of the examined grain strength properties are presented in Figure 8, whereas the results of detailed descriptive statistics are shown in Table 5.

The forces that cause rupture of the corn grain *F_RP_* were within the range (110.74–1539.94) N, when the bioyield point forces *F_BP_* were within the range (17.23–950.20) N. Stresses *S_BP_* caused by force *F_BP_* were in the range (2.94–80.4) MPa and stresses *S_RP_* were in the range (6.12–88.4) MPa. The specific energy (work) *E_mBP_* needed to induce permanent plastic deformation of grains was in the range (3.53–942.91) J/kg and the specific energy needed to induce a crack was in the range (70.70–1583.78) J/kg. The values of stiffness were in the range (3.74–265) MPa and of Young’s modulus in the range (3.59–254) MPa. The mean values of displacement *D_PI_* and force *F_PI_* were 0.119 mm and 115.96 N, respectively.

**Figure 9:**
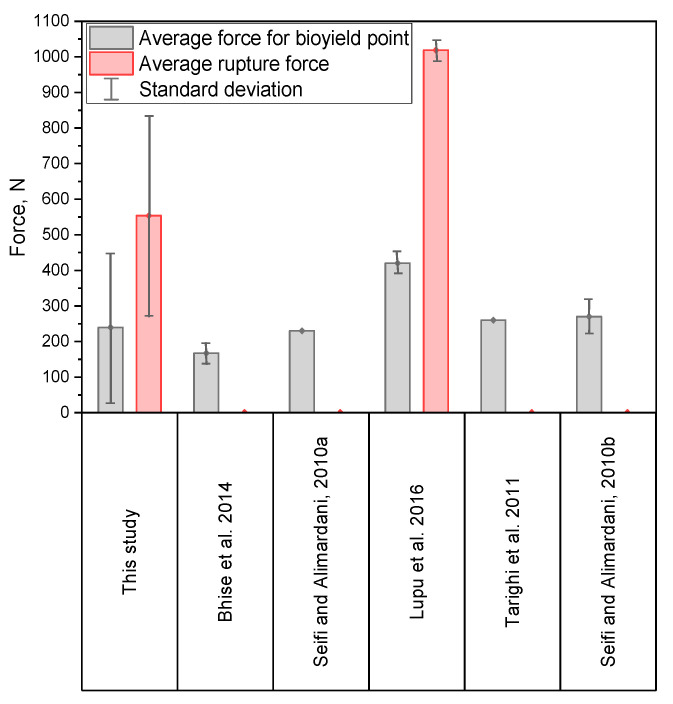
Results of average values of the forces provided in this study compared to the previous study.

In comparison with other studies in which the corn grains have a similar moisture content of 12%, the average values of forces *F_BI_* obtained in this study are similar to the results presented in [48] and [57] for the compression test at the load speed vs. 5 mm·min^−1^ (lower than in this study), higher than in the tests carried out by [45] and [47] (Figure 9). The average forces *F_BP_* and *F_RP_* in turn, were lower than those presented in [31]. This difference can be caused by a different number of samples used in calculations in [31] (*v_s_* = 50 mm·min^−1^), they used only 20 corn grains, while in this study as many as 100 grains were used. Moreover, the type of the grain and its variety could be the reason for the differences in the results presented in this study and those reported in the literature.

After a careful analysis of the literature, it can be stated that higher forces *F_BP_* and *F_RP_* should be used to compress the grain in a horizontal position (as in this study) than in a longitudinal or lateral position, as evidenced by the results presented in [53], where forces needed to break the grain in a lateral position were 116.24 and 148.86 N, and in a longitudinal position 143.39 and 186.98 N (for moisture content of grains 14.48%). Moreover, the values of energy *E_BP_* and *E_RP_* presented in [53] was lower than in this study.

**Figure 10:**
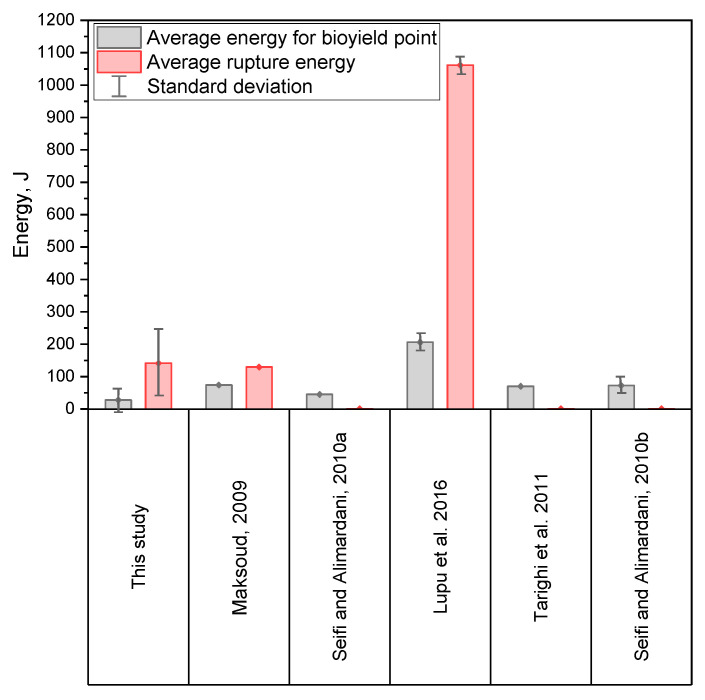
Results of average values of the energy obtained in this study compared to the previous study.

The average values of energy *E_BP_* and *E_RP_* are smaller than those provided by the literature (Figure 10). The differences may be caused by a different way of calculating the work (in studies [31,45,47,48,57], the method of determining the work (energy) of rupture has not been described) than in this study. Differences also can be caused by the way of interpretation and understanding of the initial deformation energy and the energy of grain disintegration. Summing up, the results presented in other studies [31,45,47,48,57] are in the range of forces and energy determined in this work.

When comparing the results of the research on forces and energy during pressing corn grains, it should be noted that in each of the studies which this comparison involves (Figure 9 and Figure 10, references [31,45,47,48,53,57]), a different corn variety was used, characterized by a different size and shape of grains, which also results from the selected variety of grains, a different internal structure resulting from the variety used, the cultivation area or the harvesting humidity. In each of the analyzed studies [31,45,47,48,53,57], a different test apparatus and a different value of the grain load and loading speed were used, which primarily affects the course and shape of the force-deformation curves, and also affects the accuracy of the measurements of force and displacement. In the works [31,45,47,48,53,57], a smaller number of grains was used during the tests, usually 10–20 grains, while in this work 100 grains were tested, hence significant values of the standard deviation may appear. As mentioned earlier, the process of pressing corn grains depends on the type of grain, its size and shape, crop humidity and harvesting humidity, as well as the cultivation culture. Taking into account the fact that in each study a different variety was used, the grains came from different regions with different cultivation cultures, moreover, different test methods were used, one can expect a variety of results in terms of forces and energy, which are presented in Figure 9 and Figure 10. Only in work [53], when determining energy and forces, strains, and stresses, the ASAE S368.4 [72] standard was followed, similarly to this work, and it should be stated that the results of compression forces and energy are similar in both tests (in [53] and in this study).

When analyzing the influence of individual factors, it should be indicated that the use of higher loading speeds during compression tests will reduce the accuracy of the results and may lead to a situation, in which the presence of the point of inflection and bioyield point on the force-deformation curve will not be registered and may cause the omission of the moment of grain breakage, which may result in that rather than the force corresponding to fracture. In addition, the force causing secondary agglomeration of the fractured grain particles will be indicated as the value of the destructive force.

The influence of the variety and cultivation conditions of corn grains used in the compared studies [31,45,47,48,53,57] is as follows: In the case of cultivars with a higher content of soft endosperm, the values of compressive forces and energy will be lower. Similarly, for grains of larger dimensions with a greater thickness with a more rounded shape and for grains with higher moisture, which was observed, among others, in the works [47,48,53]. The endosperm content as well as the shape and size of the grains are influenced by the type of variety used and the cultivation culture, as well as the degree of grain maturity. Therefore, there are many factors that affect the differences in the obtained results in the compared studies [31,45,47,48,53,57]. However, taking into account the dispersion of the results in this study based on the standard deviation (Figure 9 and Figure 10), it can be noted that the values reported by other researchers [31,45,47,48,53,57] fall within the range indicated in this study.

Undoubtedly, a significant scatter of the results for forces, energy, strain, stiffness or values of the apparent Young’s modulus has been found. In this case, the diversity of properties of biological materials of plant origin within one species has been confirmed. There were grains of very low compression strength for which application of a very small force led to a crack, but there were also grains of high strength which fractured under the impact of a very high force. Grain fracture under a smaller load could be caused by previous damage to its internal structure in the form of microfractures unnoticeable for the human eye due to harvest, transport or packing. The significant scatter of the results can also be caused by diversification of the grain internal structure. As previous tests of biomass grain indicate, the ratio of the seed coat thickness to the endosperm and also the structure itself (its glassiness) affect the values of destructive forces and the grain fracture propagation. [3,37,38,39,40,41]. Hence, it is necessary to conduct further tests of the impact of the grain internal structure on its mechanical properties. Development of computer micro-tomography can facilitate noninvasive tests of the grain internal structure prior to strength tests, which subsequently can contribute to the effective determination of dependencies between the grain internal structure and its mechanical properties.

Based on the results of skewness and kurtosis (Table 5) and Shapiro-Wilk test (Table 6), it was found that distributions of the analyzed grain strength values are not of normal character. In the case of *F_PI_*, *F_BP_*, and *F_RP_* forces, the Kolmogorov-Smirnov modified test showed that the tested properties can come from Weibull distribution (Table 6), though assuming the probability level to be *p* < 0.01, it cannot be ruled out that the results come from a lognormal distribution test (Table 6), and for destructive forces also from Gamma distribution. Deformation energy can be described by means of lognormal distribution, Weibull or Gamma distributions (with the exception of *E_mPI_*, see Table 6). Stiffness, apparent Young’s modulus, and rupture stresses *S_RP_* can come from lognormal, Weibull or Gamma distributions, whereas the stresses corresponding to the point of inflection and bioyield point can be described by means of Weibull and Lognormal distributions (Table 6). The distribution parameters determined for the analyzed properties are presented in Table A1.

**Figure 11:**
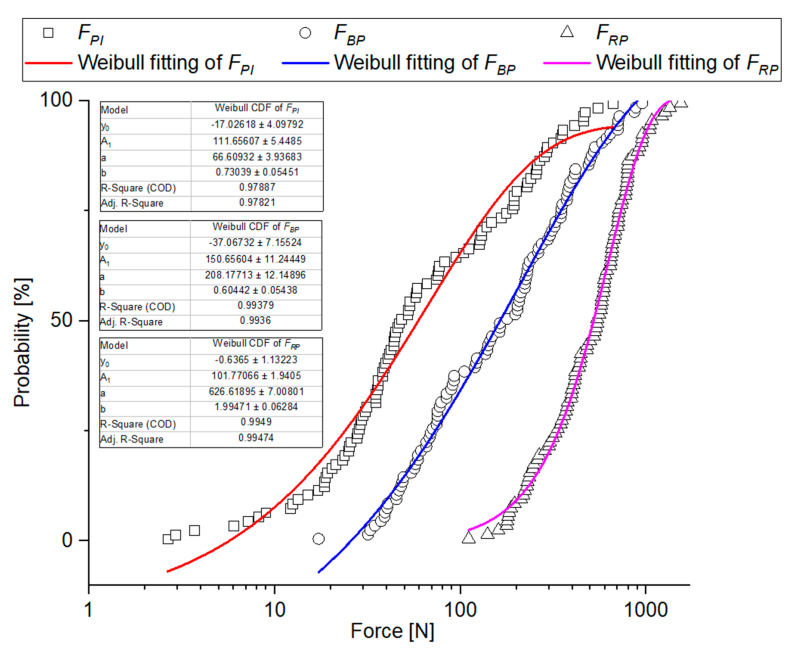
Probability plot distributions fitted to experimental values of forces.

**Figure 12:**
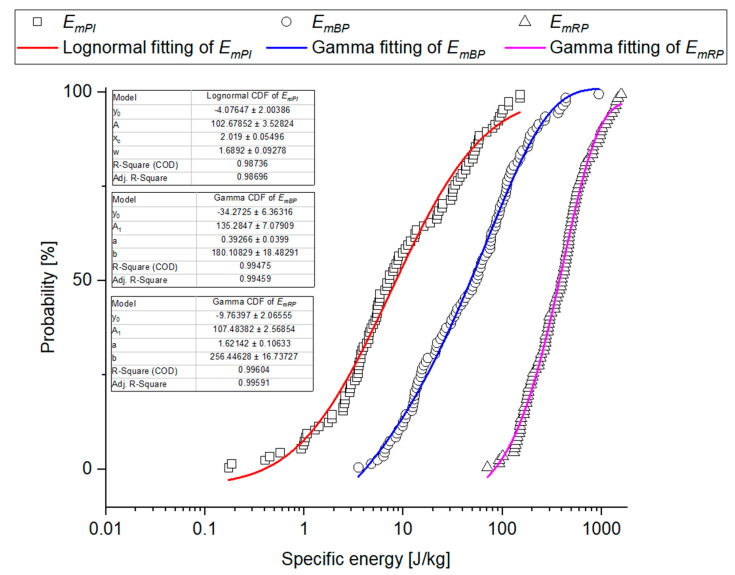
Probability plot distributions fitted to experimental values of specific energy.

**Figure 13:**
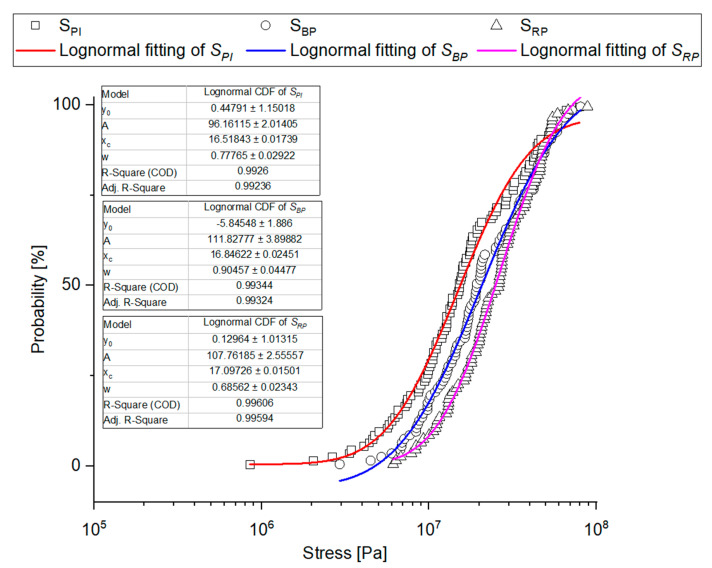
Probability plot distributions fitted to experimental values of stress.

**Figure 14:**
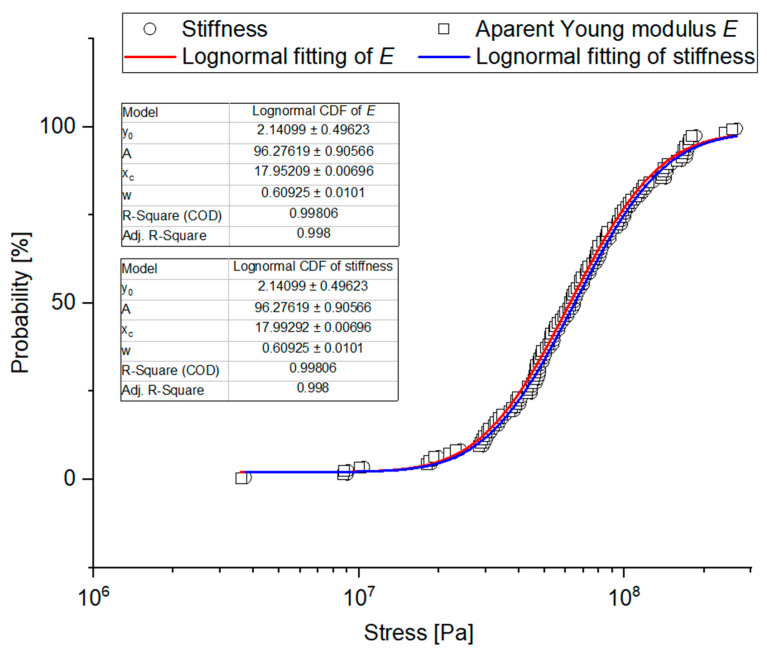
Probability plot distributions fitted to experimental values of stiffness and Young’s modulus.

Empirical charts of cumulated probability of the analyzed physical-mechanical properties were created based on the experimental data and they were provided with fitting curves for the three studied distributions: Cumulated Weibull distribution, cumulated lognormal distribution, and cumulated Gamma distribution, taking into consideration the results of Kolmogorov-Smirnov modified test. Determination coefficient R^2^ was a determinant of fitting. Table A2 shows a ranking of fitting for the analyzed cumulated distributions. Figure 11, Figure 12, Figure 13 and Figure 14 show empirical curves along with the curves of the best fitting. In the case of forces (Figure 11), it is Weibull distribution which best describes data density distribution and cumulated probability distribution. It accounts for the probability distribution of forces corresponding to the point of inflection in 97.8% *F_PI_*, in 99.4% for the forces corresponding to bioyield point *F_BP_*, and in 99.5% for the destructive forces. The cumulated Gamma distribution very well describes the grain fracture for a given level of specific energy *E_mRP_* (99.6%) and probability distribution of energy results of bioyield point *E_mBP_* (99.4%) (Figure 12). In the case of *E_mPI_*, the probability distribution is best described by the cumulated lognormal distribution (98.7%). The distribution of stress values, Young’s modulus, and stiffness is best described by the cumulated lognormal distribution (R^2^ > 0.990, Figure 13 and Figure 14).

The results are of practical significance. Most importantly, knowing the range of force, energy, and deformation is crucial for manufacturers of processing machines, as it allows preventing, e.g., uncontrolled rupture of grains during transport or classification and can be used to increase the efficiency of grinding machines through a proper selection of structural features and speed of the working units that cause the occurrence of grain damaging loads.

Grain fracture probability models provide the basis for simulation of the grinding process models and prediction of the grinding product particle size. M. Tavares’s grinding model is based on the upper-truncated log-normal probability distribution of specific fracture energy [87]. Moreover, the usefulness of Weibull distribution for material fracture probability was confirmed [59]. In work [58], it is the gamma distribution that is considered to be the best for the description of specific fracture energy values, which is consistent with the results presented in this work. The results indicate that fracture probability distributions used, e.g., for iron ore pellets [58,59] can be implemented in a description of the phenomena involved in the fracture of plant and biological materials which exhibit a significant scatter of the results.

### 3.3. Grain Size Effect

**Figure 15:**
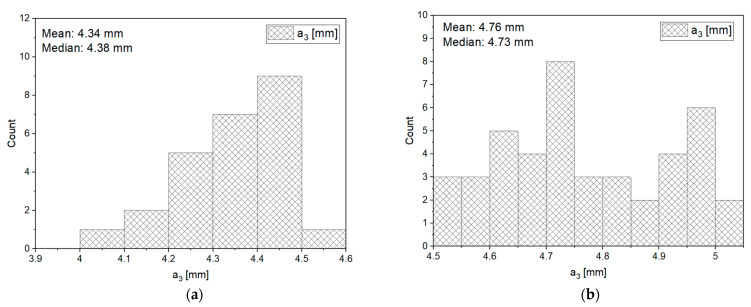

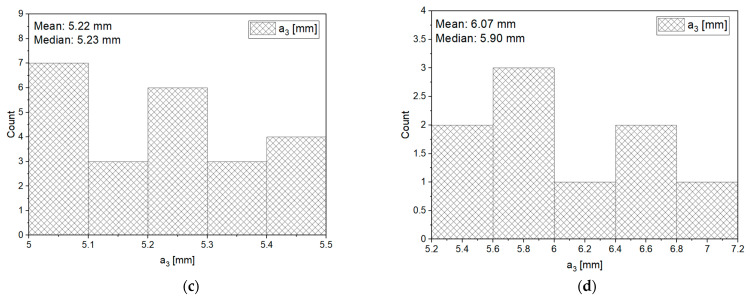
Distribution of particle sizes in the analyzed size intervals (**a**) distribution in the size range (4.0–4.5) mm, (**b**) distribution in the size range (4.5–5.0) mm, (**c**) distribution in the size range (5.0–5.5) mm, (**d**) distribution in the size range (>5.5) mm.

As proven in the previous chapter, corn grains even within one species exhibit significant dimensional diversity, which has been confirmed by significant scatters and the provided value ranges. The study was supposed to find out whether there were any dependencies and which of them were between the size of particles and forces, energy, and strength for characteristic points of the force-deformation such as point of inflection, bioyield point, and rupture point. Corn grains were divided into four dimensional fractions according to the grain thickness *a*_3_: 1—(4.0–4.5) mm, 2—(4.5–5.0) mm, 3—(5.0–5.5) mm, 4 —(>5.5) mm, for which particle size distributions along with medians and means are presented in Figure 15.

In the first step, fitted cumulated probability distributions were determined for the analyzed properties of each group. Table A3 shows the distributions obtained and their parameters. The cumulated Weibull distribution was accepted to be the best for the description of probability distribution, for energy the best one was gamma distribution, and for stress it was the cumulated lognormal distribution. Medians were determined for experimental distributions based on fitting curves. Next, the Pearson analysis of correlation between the analyzed distribution medians and the average grain thickness was performed. The Pearson coefficient assumed negative values (though these dependencies were statistically significant only for forces, energy, and stresses that cause grain fracture, Table 7) for all the cases, which proves that the values of forces, energy, and stresses decrease along with an increase in the grain thickness.

**Figure 16:**
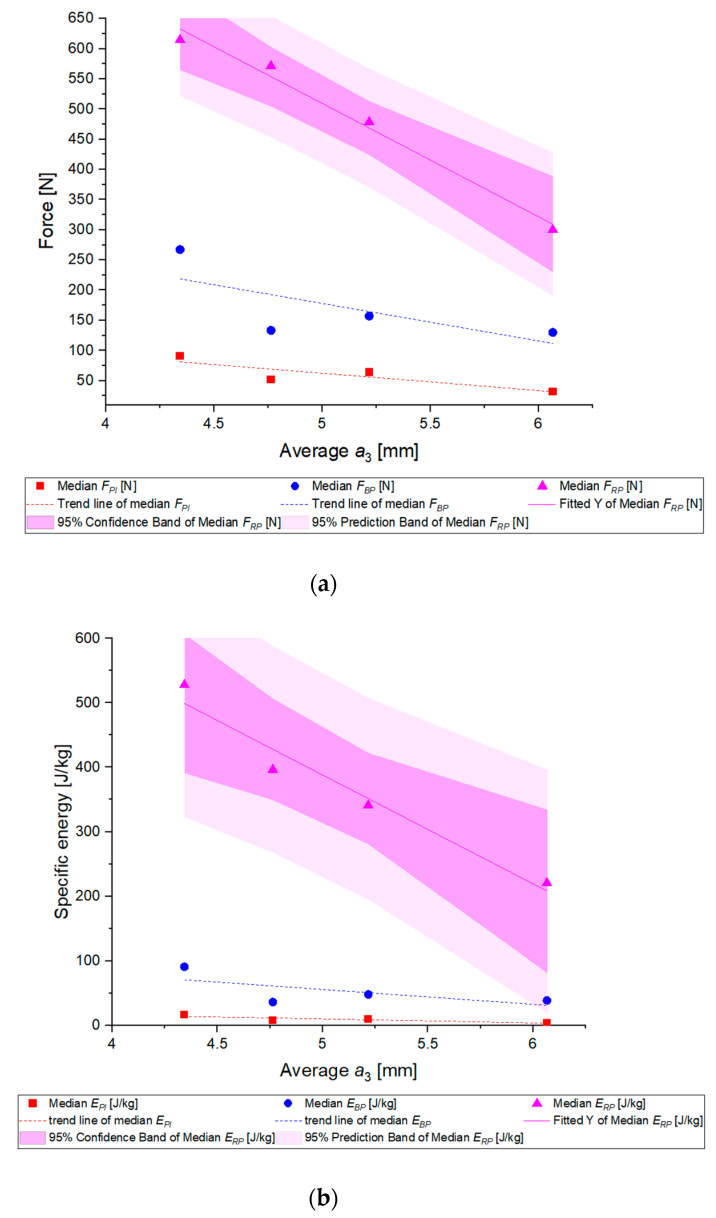

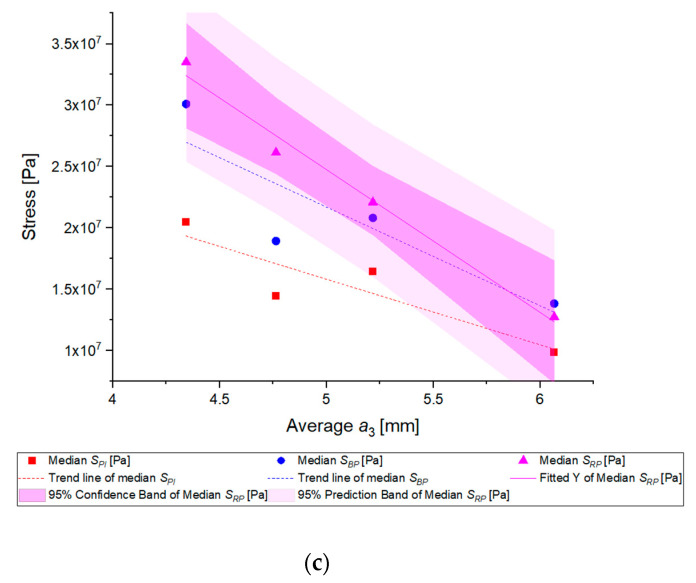
Medians of distributions of the analyzed strength properties in a function of grain mean thickness (**a**) medians of forces values distributions in a function of grain mean thickness, (**b**) medians of specific energy values distributions in a function of grain mean thickness, (**c**) medians of stresses values distributions in a function of grain mean thickness

Parameter changes along with the grain thickness are presented in Figure 16.

It is clearly seen that for the thickest grains the probability of rupture is higher under the applied load (Figure 16 and Table A3). It must also be noted that distributions of energy and deformation medians for grains with the highest thickness were the lowest, whereas for grains with the lowest thickness were the highest (Figure 16). In order to present the dependences of the analyzed parameters on the particle thickness, Figure 16 includes additional trend lines and linear regression with confidence limits for forces, energy, and stresses that induce grain rupture. The slopes of trend lines and fitting curves take negative values, so it is evident that forces, energy, and stresses corresponding to the point of inflection, bioyield point, and rupture point decrease along with the grain height. The presented results confirm the hypotheses and assumptions for grains of wheat, rice or non-biological materials, e.g., iron ore pellets discussed in earlier works [4,59,77,78], that the size of particle has an impact on the values of forces, stresses, and deformation energy. Basically, the occurrence of a negative correlation between grain thickness and grinding energy has been confirmed, which is presented in a work devoted to wheat grains [4].

The presented results also seem to confirm the conclusions provided in work [59] concerning the increasing stiffness for decreasing grain dimensions. In the case of biological materials such as grains, an increase in force and energy along with size reduction can be caused by the grain structure, as well as the smaller porosity of grains of smaller size. The ratio of thickness of the softer endosperm layer to the harder seed cover layer can also have an impact.

### 3.4. Limitations and Advantages

Undoubtedly, the fact that a population of corn grains of stabilized moisture parameters has been tested and known from the literature distribution parameters, providing a good description of experimental empirical distributions of values for the corn grain selected physical-mechanical properties have been determined, is an advantage of this study. Unlike in other works [31,47,57], the experiment and determination of mechanical properties were carried out according to the ASAE S368.4 standard [73], hence, it can be assumed that the determined values of work, energy, Young’s modulus, and destructive stress provide a good description of the corn grain mechanical properties. Moreover, the values of mechanical properties were determined for the point of inflection which is rather rare in the literature. One of the limitations of the study carried out is the lack of information on the humidity of the corn grain harvest, which indicates the degree of grain maturity, which in turn may affect the hardness and strength properties of the grains. Lack of identification of the corn grain internal structure, which has a significant influence on the rupture process, can also be considered to be a certain limitation. Another drawback of this study is the application of only the most known result distributions.

## 4. Conclusions

The mean values and size ranges characterizing the shape and size of corn grains have been determined in this work, as well as the mechanical properties and distributions of probability for a given property to occur. The diversity of biomass grains within one species has been confirmed, which is unequivocally indicated by significant scatters of results for the analyzed sample of 100 grains.

Based on statistical analyses of the investigated values of the grain size and shape it has been found that the distributions of experimental data can be described with good fitting by means of known and commonly used distributions: Normal, gamma, lognormal, and Weibull distributions. For forces, energy, stresses, stiffness, and Young’s modulus the distributions of values cannot be described by means of a normal distribution. The empirical distribution of destructive force occurrence probability is best described by the cumulated Weibull distribution, energy by cumulated Gamma distributions, and for stiffness, stress, and apparent Young’s modulus by cumulated lognormal distributions. Determined distributions of rupture occurrence probability (that is, distributions for forces, energy, and stresses that cause grain rupture) and their parameters are indispensable in a design of processing machines intended for materials of plant origin such as grains, e.g., grinders, conveyors, classifiers, etc. The obtained distributions are also important in the numerical modeling of grinding processes as the models are often based on breakage probability distributions.

The results of this study have confirmed the assumption that forces, energy, and stresses that cause grain deformation and rupture depend on the grain size, and more accurately, the grain thickness. A decrease in these parameters along with the thickness increase was observed.

Further tests should be focused on the relationship between the grain internal structure and mechanical properties, which can be supported by developing methods for structure recognition and analysis using non-invasive tests that would allow analyzing the impact of the grain layer thickness on its behavior under the influence of destructive forces. Further research should also include the determination of strength properties of corn grains under dynamic conditions, which due to the nature of the applied load may differ significantly from the properties determined under static conditions. Additionally, such tests will better reflect the real nature of the loads to which the grains are subjected during mechanical processing.

## Figures and Tables

**Table 1:** Tested probability distributions.

Distribution	Mathematical Model	
Normal	$P=\frac{1}{x\sqrt{2\pi \sigma^{2}}}\exp\left[-\frac{{\left(x-\mu \right)}^{2}}{2\sigma^{2}}\right]$	(18)
Lognormal	$P=\frac{1}{x\sqrt{2\pi \sigma^{2}}}\exp\left[-\frac{{\left(\ln(x)-\mu \right)}^{2}}{2\sigma^{2}}\right]$	(19)
Weibull	$P=\frac{\beta }{\alpha^{\beta }}x^{\beta -1}\exp\left[-{\left(\frac{x}{\alpha }\right)}^{\beta }\right]$	(20)
Gamma	$P=\frac{1}{\Gamma (\alpha )\sigma^{\alpha }}x^{\alpha -1}\exp\left[-\frac{x}{\sigma }\right]$	(21)

**Table 2:** Cumulated probability distributions used in the analysis.

Cumulative Distribution Function	Mathematical Model	
Lognormal	$P_{CDF}=y_{0}+A\int_{0}^{x}\frac{1}{\sqrt{2\pi wt}}e^{-\frac{{\left(\ln(t)-x_{c}\right)}^{2}}{2w^{2}}}dt$	(23)
Gamma	$P_{CDF}=y_{0}+\frac{A_{1}}{b^{a}\Gamma (a)}\int_{0}^{x}t^{a-1}e^{-\frac{t}{b}}dt$	(24)
Weibull	$P_{CDF}=y_{0}+A_{1}\int_{0}^{x}ba^{-b}t^{b-1}e^{-{\left(\frac{t}{a}\right)}^{b}}dt=y_{0}+A_{1}\left(1-e^{-{\left(\frac{x}{a}\right)}^{b}}\right)$	(25)

**Table 3:** Results of statistical analysis of the examined selected physical properties of corn grains.

Parameter	*a*_1_ [mm]	*a*_2_ [mm]	*a*_3_ [mm]	*f* [–]	*R_a_* [–]	*m* [g]	*V_g_* [mm^3^]	*V_t_* [mm^3^]	kV [–]	*D_E_* [mm]
Average	10.65	7.85	4.88	0.698	0.742	0.302	213.40	251.67	0.858	7.806
Median	10.72	7.90	4.78	0.689	0.742	0.300	211.54	250.00	0.845	7.816
Standard deviation	0.91	0.82	0.52	0.057	0.094	0.052	35.62	42.97	0.123	0.465
Skewness	−1.15	−1.50	1.44	1.267	−0.510	−0.332	0.08	−0.33	0.230	−0.746
Kurtosis	2.50	7.40	3.30	4.324	4.392	0.052	1.31	0.05	4.133	1.147
Minimum	7.24	3.40	4.00	0.521	0.307	0.140	100.70	116.67	0.336	6.062
Maximum	12.62	9.76	7.03	0.934	1.036	0.400	335.01	333.33	1.292	8.603
Range	5.38	6.36	3.03	0.413	0.729	0.260	234.31	216.67	0.956	2.540
Coefficient of variation	8.53	10.48	10.75	8.125	12.626	17.073	16.69	17.07	14.384	5.957

**Table 4:** Results of the distribution of goodness-of-fit tests.

Property	Shapiro-Wilk Test		Kolmogorov-Smirnov Modified Test					
	Normal		Lognormal		Weibull		Gamma	
	Statistic	*p*-Value	Statistic	*p*-Value	Statistic	*p*-Value	Statistic	*p*-Value
*a*_1_ [mm]	0.92149	1.68911 × 10^−5^	0.16195	≤0.01	0.0944	>0.1	0.15401	≤0.005
*a*_2_ [mm]	0.90576	2.66544 × 10^−6^	0.08903	0.0495	0.05821	>0.1	0.07293	>0.25
*a*_3_ [mm]	0.90579	2.67481 × 10^−6^	0.08664	0.06452	0.15801	0.02197	0.09354	0.0459
f [–]	0.88855	4.24999 × 10^−7^	0.12545	≤0.01	0.18801	≤0.01	0.13035	≤0.005
Ra [–]	0.94218	2.62455 × 10^−4^	0.09527	0.03379	0.09752	>0.1	0.08562	0.09943
*m* [g]	0.98115	0.16318	-	-	-	-	-	-
*V_g_* [mm^3^]	0.98671	0.41869	-	-	-	-	-	-
*V_t_* [mm^3^]	0.98115	0.16318	-	-	-	-	-	-
kV [–]	0.91001	4.31216 × 10^−6^	0.12653	≤0.01	0.17343	≤0.01	0.12976	≤0.005
*D_E_* [mm]	0.96098	0.00472	0.07756	0.14271	0.08834	>0.1	0.07943	0.15827

**Table 5:** Results of statistical analysis of the examined selected physical properties of corn grains.

Parameter	*F_PI_* [N]	*F_BP_* [N]	*F_RP_* [N]	*D_PI_* [mm]	*D_BP_* [mm]	*D_RP_* [mm]	*Em_PI_* [J/kg]	*Em_BP_* [J/kg]	*Em_RP_* [J/kg]	*S_PI_* [Pa]	*S_BP_* [Pa]	*S_RP_* [Pa]	*E* [Pa]	*Stiffness* [Pa]
Mean	115.96	239.75	553.80	0.119	0.227	0.564	23.77	92.65	477.10	2.11 × 10^7^	2.60 × 10^7^	2.83 × 10^7^	7.50 × 10^7^	7.81 × 10^7^
Standard Deviation	135.81	215.63	286.71	0.061	0.108	0.204	33.45	129.75	349.79	1.68 × 10^7^	1.76 × 10^7^	1.57 × 10^7^	4.93 × 10^7^	5.13 × 10^7^
Skewness	1.77	1.36	0.83	0.777	1.512	0.925	2.01	3.65	1.37	1.29	0.98	0.94	1.29	1.29
Kurtosis	2.94	1.37	0.82	−0.262	3.445	0.990	3.77	18.80	1.36	1.04	0.15	0.98	1.75	1.75
Coefficient of Variation	1.17	0.90	0.52	0.513	0.477	0.361	1.41	1.40	0.73	0.80	0.68	0.56	0.66	0.66
Minimum	2.65	17.23	110.74	0.020	0.087	0.256	0.17	3.53	70.70	8.52 × 10^5^	2.94 × 10^6^	6.12 × 10^6^	3.59 × 10^6^	3.74 × 10^6^
Median	47.38	170.84	544.74	0.104	0.208	0.530	7.03	54.46	396.43	1.52 × 10^7^	1.93 × 10^7^	2.62 × 10^7^	6.22 × 10^7^	6.48 × 10^7^
Maximum	659.84	950.20	1539.94	0.265	0.681	1.304	150.48	942.91	1583.78	7.93 × 10^7^	8.04 × 10^7^	8.84 × 10^7^	2.54 × 10^8^	2.65 × 10^8^
Range	657.19	932.97	1429.20	0.245	0.594	1.048	150.30	939.38	1513.08	7.84 × 10^7^	7.75 × 10^7^	8.23 × 10^7^	2.50 × 10^8^	2.61 × 10^8^

**Table 6:** Results of distribution of the goodness-of-fit tests.

Property	Shapiro-Wilk Test		Kolmogorov-Smirnov Modified Test					
	Normal		Lognormal		Weibull		Gamma	
	Statistic	*p*-Value	Statistic	*p*-Value	Statistic	*p*-Value	Statistic	*p*-Value
*F_PI_* [N]	0.75967	1.69986 × 10^−11^	0.08888	0.04987	0.14577	0.05921	0.16277	≤0.005
*F_BP_* [N]	0.84484	7.67875 × 10^−9^	0.09377	0.03757	0.11384	>0.1	0.12207	≤0.005
*F_RP_* [N]	0.94883	6.95722 × 10^−4^	0.09072	0.04526	0.06169	>0.1	0.06076	>0.25
*E_mPI_* [J/kg]	0.67974	1.86462 × 10^−13^	0.07698	0.14929	0.12495	>0.1	0.15474	≤0.005
*E_mBP_* [J/kg]	0.62964	1.63203 × 10^−14^	0.08057	0.10824	0.06883	>0.1	0.08864	0.07781
*E_mRP_* [J/kg]	0.85688	2.14812 × 10^−8^	0.05841	>0.15	0.07434	>0.1	0.06492	>0.25
*S_PI_* [Pa]	0.85758	2.28423 × 10^−8^	0.06069	>0.15	0.12295	>0.1	0.1123	0.0053
*S_BP_* [Pa]	0.89738	1.0673 × 10^−6^	0.0735	>0.15	0.12692	>0.1	0.11189	0.00563
*S_RP_* [Pa]	0.9313	5.90074 × 10^−5^	0.05331	>0.15	0.07236	>0.1	0.05269	>0.25
*E* [Pa]	0.89642	9.63659 × 10^−7^	0.07299	>0.15	0.07262	>0.1	0.05103	>0.25
*Stiffness* [Pa]	0.89642	9.63659 × 10^−7^	0.07299	>0.15	0.07262	>0.1	0.05103	>0.25

**Table 7:** Results of analysis of the correlation between grain thickness and medians of cumulated probability distributions of the analyzed mechanical properties of maize grains, * means significant correlations for which *p* < 0.05.

		Median *F_PI_*	Median *F_BP_*	Median *F_RP_*	Median *E_mPI_*	Median *E_mBP_*	Median *E_mRP_*	Median *S_PI_*	Median *S_BP_*	Median *S_RP_*
Average *a*_3_	r-Pearson’s	−0.86507	−0.70918	−0.99289 *	−0.87089	−0.66334	−0.97821 *	−0.8959	−0.87365	−0.9926 *
	*p*-Value	0.13493	0.29082	0.00711	0.12911	0.33666	0.02179	0.1041	0.12635	0.0074

## Data Availability

The data presented in this study are openly available in FigShare at https://doi.org/10.6084/m9.figshare.13691239.v1 (accessed on 2 February 2021).

## Appendix A

**Table A1:** Parameters of probability distributions for strength properties.

Parameter	Distribution					
	Lognormal		Weibull		Gamma	
	*µ*	*σ*	*α*	*β*	*α*	*σ*
*F_PI_* [N]	-	-	109.1439	0.89281	-	-
*F_BP_* [N]	-	-	253.61107	1.16228	-	-
*F_RP_* [N]	-	-	627.11731	2.06287	3.64698	151.85166
*E_mPI_* [J/kg]	2.17009	1.54562	18.73184	0.71195	-	-
*E_mBP_* [J/kg]	3.82259	1.23579	84.44912	0.85467	0.83563	110.87089
*E_mRP_* [J/kg]	5.91986	0.71669	531.83095	1.48228	2.16927	219.93337
*S_PI_* [Pa]	16.5451	0.8459	2.3033 × 10^7^	1.33441	-	-
*S_BP_* [Pa]	16.83799	0.71216	2.91069 × 10^7^	1.5688	-	-
*S_RP_* [Pa]	16.99646	0.58967	3.19944 × 10^7^	1.92147	3.27416	8,630,478.29
*E* [Pa]	17.90752	0.73007	8.39823 × 10^7^	1.61551	2.3764	3.15428 × 10^7^
*Stiffness* [Pa]	17.94835	0.73007	8.74816 × 10^7^	1.61551	2.3764	3.28571 × 10^7^

**Table A2:** Coefficient of R^2^ determination of cumulated Weibull, gamma, and lognormal distributions.

Parameter	*R*^2^ of Distributions		
	Lognormal	Weibull	Gamma
*F_PI_* [N]	-	0.979	-
*F_BP_* [N]	-	0.994	-
*F_RP_* [N]	-	0.995	0.993
*E_mPI_* [J/kg]	0.987	0.982	-
*E_mBP_* [J/kg]	0.992	0.994	0.995
*E_mRP_* [J/kg]	0.9955	0.9958	0.996
*S_PI_* [Pa]	0.992	0.987	-
*S_BP_* [Pa]	0.993	0.991	-
*S_RP_* [Pa]	0.9959	0.9956	0.9958
*E* [Pa]	0.998	0.995	0.997
*Stiffness* [Pa]	0.998	0.995	0.997

**Table A3:** Cumulated distributions of probability of the occurrence of forces, energy, and stresses while compressing corn grains and their parameters.

Weibull fitting		4.0–4.5 mm	4.5–5.0 mm	5.0–5.5 mm	>5.5 mm
	y_0_	–30.54 ± 23.64	−15.92 ± 6.35	−4.21 ± 6.76	−4.44 ± 14.44
	A_1_	222.15 ± 193.17	106.44 ± 7.4	96.81 ± 9.45	91.08 ± 16.39
	a	777.77 ± 2433.01	53.85 ± 4.37	78.06 ± 10.09	33.61 ± 4.10
	b	0.37 ± 0.19	0.87 ± 0.10	0.99 ± 0.18	1.91 ± 0.76
	µ	3218.20 ± 15,088.27	57.59 ± 4.76	78.51 ± 12.82	29.82 ± 3.60
	σ	11,482.90 ± 64,018.93	66.03 ± 11.30	79.55 ± 24.71	16.26 ± 6.10
	R^2^	0.988	0.975	0.958	0.956
Weibull fitting		4.0–4.5 mm	4.5–5.0 mm	5.0–5.5 mm	>5.5 mm
	y_0_	3.33 ± 3.95	−1884.40 ± 22,431.29	−5.35 ± 4.69	−238.77 ± 8193.17
	A_1_	97.18 ± 8.91	2072.44 ± 22,682.30	100.75 ± 6.65	3151.18 ± 1,590,007.82
	a	380.59 ± 33.86	7.73 × 10^−4^ ± 0.08	189.18 ± 10.25	1.80 × 10^9^ ± 7.89 × 10^12^
	b	1.19 ± 0.18	0.083 ± 0.43	1.22 ± 0.14	0.14 ± 7.54
	µ	358.67 ± 42.16	476,075.22 ± 2.69 × 10^7^	177.19 ± 10.73	9.47 × 10^12^ ± 4.86 × 10^16^
	σ	302.24 ± 75.02	8.37 × 10^8^ ± 8.28 × 10^10^	145.91 ± 21.34	5.63 × 10^14^ ± 3.02 × 10^18^
	R ^2^	0.986	0.990	0.989	0.971
Weibull fitting		4.0–4.5 mm	4.5–5.0 mm	5.0–5.5 mm	>5.5 mm
	y_0_	0.08 ± 3.30	5.26 ± 2.15	−0.77 ± 3.71	−6.38 ± 22.08
	A_1_	102.84 ± 7.15	99.72 ± 4.84	99.75 ± 5.39	107.79 ± 40.40
	a	771.20 ± 35.71	692.45 ± 18.83	554.65 ± 15.02	349.56 ± 74.61
	b	1.79 ± 0.19	2.68 ± 0.20	2.30 ± 0.22	1.96 ± 1.03
	µ	686.07 ± 33.80	615.64 ± 16.27	491.37 ± 13.36	309.92 ± 67.81
	σ	397.05 ± 51.55	247.44 ± 19.18	226.48 ± 19.94	165.14 ± 100.06
	R ^2^	0.991	0.983	0.990	0.941
Gamma fitting		4.0–4.5 mm	4.5–5.0 mm	5.0–5.5 mm	>5.5 mm
	y_0_	−46.42 ± 32.56	−22.10 ± 9.63	−8.06 ± 6.87	4.87 ± 4.71
	A_1_	657.19 ± 605,661.04	112.84 ± 10.37	104.16 ± 9.28	83.63 ± 5.88
	a	0.18 ± 0.08	0.48 ± 0.10	0.63 ± 0.14	6.50 ± 2.07
	b	1,282,758.94 ± 6.68 × 10^9^	19.70 ± 4.28	22.07 ± 6.79	0.61 ± 0.20
	µ	227,235.93 ± 1.18 × 10^9^	9.52 ± 0.74	13.96 ± 2.06	3.98 ± 0.19
	σ	539,897.14 ± 2.81 × 10^9^	13.69 ± 1.70	17.55 ± 3.79	1.56 ± 0.27
	R ^2^	0.982	0.983	0.979	0.984
Gamma fitting		4.0–4.5 mm	4.5–5.0 mm	5.0–5.5 mm	>5.5 mm
	y_0_	2.10 ± 4.44	−498.77 ± 697.04	−14.30 ± 18.17	−24.87 ± 45.03
	A_1_	95.01 ± 6.68	599.65 ± 699.21	146.31 ± 55.33	138.26 ± 70.04
	a	0.89 ± 0.19	0.06 ± 0.07	0.53 ± 0.25	0.55 ± 0.51
	b	152.22 ± 42.16	270.98 ± 92.32	254.23 ± 305.36	120.87 ± 165.61
	µ	135.74 ± 13.18	14.95 ± 15.18	133.94 ± 102.15	66.10 ± 35.26
	σ	143.74 ± 25.64	63.64 ± 23.59	184.53 ± 180.33	89.38 ± 83.06
	R ^2^	0.984	0.99201	0.976	0.986
Gamma fitting		4.0–4.5 mm	4.5–5.0 mm	5.0–5.5 mm	>5.5 mm
	y_0_	−16.86 ± 10.24	0.003 ± 3.28	−3.80 ± 5.29	5.56 ± 0
	A_1_	154.87 ± 34.96	97.75 ± 4.30	100.96 ± 6.69	88.85 ± 14.91
	a	0.70 ± 0.20	2.67 ± 0.39	2.95 ± 0.61	3.27 ± 1.81
	b	1684.99 ± 1089.80	166.08 ± 24.23	123.39 ± 24.87	74.91 ± 56.93
	µ	1177.84 ± 445.69	442.98 ± 11.78	364.45 ± 12.41	244.86 ± 57.38
	σ	1408.78 ± 718.86	271.23 ± 20.47	212.06 ± 21.49	135.43 ± 66.26
	R ^2^	0.993	0.988	0.991	0.912
Lognormal fitting		4.0–4.5 mm	4.5–5.0 mm	5.0–5.5 mm	>5.5 mm
	y_0_	−14.48 ± 12.26	−1.06 ± 2.56	7.80 ± 2.01	−1.80 ± 15.32
	A	178.37 ± 66.38	93.80 ± 3.63	85.79 ± 3.50	104.40 ± 24.32
	x_c_	17.40 ± 0.52	16.41 ± 0.03	16.62 ± 0.04	16.11 ± 0.12
	w	1.60 ± 0.55	0.68 ± 0.05	0.62 ± 0.06	0.61 ± 0.21
	µ	1.31 × 10^8^ ± 1.83 × 10^8^	1.69 × 10^7^ ± 687,179.05	2.01 × 10^7^ ± 1,228,354.58	1.19 × 10^7^ ± 1,666,522.57
	σ	4.56 × 10^8^ ± 1.07 × 10^9^	1.30 × 10^7^ ± 1,577,012.61	1.38 × 10^7^ ± 2,290,183.04	8,001,674.74 ± 4,164,865.25
	R^2^	0.991	0.988	0.985	0.982
Lognormal fitting		4.0–4.5 mm	4.5–5.0 mm	5.0–5.5 mm	>5.5 mm
	y_0_	−17.44 ± 35.46	−21.7 ± 11.14	5.57 ± 2.26	−8.10 ± 17.38
	A	1137.62 ± 7596.58	132.59 ± 18.83	87.68 ± 3.58	118.46 ± 32.16
	x_c_	21.43 ± 16.33	16.64 ± 0.08	16.84 ± 0.03	16.46 ± 0.09
	w	2.70 ± 5.12	1.14 ± 0.20	0.51 ± 0.05	0.58 ± 0.21
	µ	7.65 × 10^10^ ± 2.30 × 10^12^	3.21 × 10^7^ ± 6,842,766.15	2.35 × 10^7^ ± 1,001,842.68	1.66 × 10^7^ ± 2,468,284.52
	σ	2.89 × 10^12^ ± 1.27 × 10^14^	5.22 × 10^7^ ± 2.72 × 10^7^	1.30 × 10^7^ ± 1,763,464.72	1.04 × 10^7^ ± 5,738,377.74
	R ^2^	0.984	0.990	0.986	0.989
Lognormal fitting		4.0–4.5 mm	4.5–5.0 mm	5.0–5.5 mm	>5.5 mm
	y_0_	−10.90 ± 22.68	1.52 ± 1.29	−2.03 ± 4.01	−20.51 ± 49.56
	A	1399.77 ± 11,973.53	105.08 ± 3.30	102.94 ± 6.57	129.95 ± 73.33
	x_c_	20.65 ± 14.10	17.13 ± 0.02	16.90 ± 0.045	16.28 ± 0.23
	w	1.94 ± 3.84	0.58 ± 0.03	0.69 ± 0.069	0.70 ± 0.48
	µ	6.15 × 10^9^ ± 1.32 × 10^11^	3.27 × 10^7^ ± 974,810.58	2.78 × 10^7^ ± 1,791,511.93	1.51 × 10^7^ ± 3,316,183.17
	σ	4.01 × 10^10^ ± 1.17 × 10^12^	2.08 × 10^7^ ± 1,712,189.50	2.18 × 10^7^ ± 3,850,342.57	1.20 × 10^7^ ± 1.26 × 10^7^
	R^2^	0.972	0.997	0.989	0.985

## References

[1] Kovács Á., Kerényi G. (2019). Physical Characteristics and Mechanical Behaviour of Maize Stalks for Machine Development. Int. Agrophysics.

[2] Wiercioch M., Niemiec A., Roma L. (2008). The impact of wheat seeds size on energy consumption of their grinding process. Inzynieria Rol..

[3] Werechowska M. (2014). Some Physical Properties of Cereal Grain and Energy Consumption of Grinding. Agric. Eng..

[4] Dziki D., Laskowski J. (2003). Influence of Wheat Kernel Geometrical Properties on the Mechanical Properties and Grinding Ability. Acta Agrophys..

[5] Kruszelnicka W., Bałdowska-Witos P., Kasner R., Flizikowski J., Tomporowski A., Rudnicki J. (2019). Evaluation of Emissivity and Environmental Safety of Biomass Grinders Drive. Przem. Chem..

[6] Atashbar N.Z., Labadie N., Prins C. (2018). Modelling and Optimisation of Biomass Supply Chains: A Review. Int. J. Prod. Res..

[7] Jewiarz M., Wróbel M., Mudryk K., Szufa S. (2020). Impact of the Drying Temperature and Grinding Technique on Biomass Grindability. Energies.

[8] Bembenek M. (2020). Exploring Efficiencies: Examining the Possibility of Decreasing the Size of the Briquettes Used as the Batch in the Electric Arc Furnace Dust Processing Line. Sustainability.

[9] Hryniewicz M., Bembenek M., Janewicz A., Kosturkiewicz B. (2015). Brykietowanie materiałów drobnoziarnistych w prasach walcowych z niesymetrycznym układem zagęszczania. Przem. Chem..

[10] Iqbal Y., Lewandowski I., Weinreich A., Wippel B., Pforte B., Hadai O., Tryboi O., Spöttle M., Peters D. (2016). Maximising the Yield of Biomass from Residues of Agricultural Crops and Biomass from Forestry.

[11] Chhabra N., Kaur A. (2017). Studies on Physical and Engineering Characteristics of Maize, Pearl Millet and Soybean. J. Pharmacogn. Phytochem..

[12] Kruszelnicka W., Wróbel M., Jewiarz M., Szlęk A. (2020). Study of Physical Properties of Rice and Corn Used for Energy Purposes. Proceedings of the Renewable Energy Sources: Engineering, Technology, Innovation.

[13] Chel-Guerrero L., Parra-Pérez J., Betancur-Ancona D., Castellanos-Ruelas A., Solorza-Feria J. (2015). Chemical, Rheological and Mechanical Evaluation of Maize Dough and Tortillas in Blends with Cassava and Malanga Flour. J. Food Sci. Technol..

[14] Li Y., Qin T., Chen J., Zhao Z. (2011). Experiments and Analysis on Mechanical Property of Corn Stalk Reciprocating Cutting. Trans. Chin. Soc. Agric. Eng..

[15] Moya M., Aguado P.J., Ayuga F. (2013). Mechanical Properties of Some Granular Agricultural Materials Used in Silo Design. Int. Agrophysics.

[16] Flizikowski J.B., Kruszelnicka W., Tomporowski A., Mrozinski A. (2019). A Study of Operating Parameters of a Roller Mill with a New Design. AIP Conf. Proc..

[17] Kowalczyk-Jusko A., Kowalczuk J., Szmigielski M., Marczuk A., Jozwiakowski K., Zarajczyk K., Maslowski A., Slaska-Grzywna B., Sagan A., Zarajczyk J. (2015). Quality of biomass pellets used as fuel or raw material for syngas production. Przemy. Chem..

[18] Zając G., Węgrzyn A. (2008). Analysis of Work Parameters Changes of Diesel Engine Powered with Diesel Fuel and Faee Blends. Eksploat. Niezawodn.-Maint. Reliab..

[19] Tomporowski A., Flizikowski J., Kruszelnicka W. (2017). A new concept of roller-plate mills. Przem. Chem..

[20] Dabbour M.I., Bahnasawy A., Ali S., El-Haddad Z. (2015). Grinding Parameters and Their Effects on the Quality of Corn for Feed Processing. J. Food Process. Technol..

[21] Kaczmarczyk J., Grajcar A. (2018). Numerical Simulation and Experimental Investigation of Cold-Rolled Steel Cutting. Mater. Basel Switz..

[22] Kaczmarczyk J. (2019). Modelling of Guillotine Cutting of a Cold-Rolled Steel Sheet. Materials.

[23] Tomporowski A., Flizikowski J., Wełnowski J., Najzarek Z., Topoliński T., Kruszelnicka W., Piasecka I., Śmigiel S. (2018). Regeneration of Rubber Waste Using an Intelligent Grinding System. Przem. Chem..

[24] Mannheim V. (2011). Empirical and Scale-up Modeling in Stirred Ball Mills. Chem. Eng. Res. Des..

[25] Tomporowski A., Flizikowski J., Kasner R., Kruszelnicka W. (2017). Environmental Control of Wind Power Technology. Rocz. Ochr. Śr..

[26] Flizikowski J., Piasecka I., Kruszelnicka W., Tomporowski A., Mroziński A. (2018). Destruction Assessment of Wind Power Plastics Blade. Polimery.

[27] Jachimowski R., Szczepanski E., Klodawski M., Markowska K., Dabrowski J. (2018). Selection of a Container Storage Strategy at the Rail-Road Intermodal Terminal as a Function of Minimization of the Energy Expenditure of Transshipment Devices and CO_2_ Emissions. Rocz. Ochr. Srodowiska.

[28] Korczewski Z., Rudnicki J., Salvatore F., Broglia R., Muscari R. (2015). An Energy Approach to the Fatigue Life of Ship Propulsion Systems. Proceedings of the VI International Conference on Computational Methods in Marine Engineering.

[29] González-Montellano C., Fuentes J.M., Ayuga-Téllez E., Ayuga F. (2012). Determination of the Mechanical Properties of Maize Grains and Olives Required for Use in DEM Simulations. J. Food Eng..

[30] Dobrzański B., Stępniewski A. (2013). Physical Properties of Seeds in Technological Processes. Adv. Agrophys. Res..

[31] Lupu M.I., Pădureanu V., Canja C.M., Măzărel A. (2016). The Effect of Moisture Content on Grinding Process of Wheat and Maize Single Kernel. IOP Conf. Ser. Mater. Sci. Eng..

[32] Tumuluru J.S., Tabil L.G., Song Y., Iroba K.L., Meda V. (2014). Grinding Energy and Physical Properties of Chopped and Hammer-Milled Barley, Wheat, Oat, and Canola Straws. Biomass Bioenergy.

[33] Pandiselvam R., Thirupathi V., Mohan S. (2015). Engineering Properties of Rice. Agric. Eng..

[34] Warechowska M., Warechowski J., Skibniewska K.A., Siemianowska E., Tyburski J., Aljewicz M.A. (2016). Environmental factors influence milling and physical properties and flour size distribution of organic spelt wheat. Tech. Sci..

[35] Dziki D. (2007). Ocena Energochłonności Rozdrabniania Ziarna Pszenicy Poddanego Uprzednio Zgniataniu. Inż. Rol..

[36] Dziki D., Cacak-Pietrzak G., Miś A., Jończyk K., Gawlik-Dziki U. (2014). Influence of Wheat Kernel Physical Properties on the Pulverizing Process. J. Food Sci. Technol..

[37] Zeng Y., Jia F., Xiao Y., Han Y., Meng X. (2019). Discrete Element Method Modelling of Impact Breakage of Ellipsoidal Agglomerate. Powder Technol..

[38] Sadeghi M., Araghi H.A., Hemmat A. (2010). Physico-Mechanical Properties of Rough Rice (*Oryza Sativa* L.) Grain as Affected by Variety and Moisture Content. Agric. Eng. Int. CIGR J..

[39] Cao W., Nishiyama Y., Koide S. (2004). Physicochemical, Mechanical and Thermal Properties of Brown Rice Grain with Various Moisture Contents. Int. J. Food Sci. Technol..

[40] Greffeuille V., Abecassis J., Barouh N., Villeneuve P., Mabille F., Bar L’Helgouac’h C., Lullien-Pellerin V. (2007). Analysis of the Milling Reduction of Bread Wheat Farina: Physical and Biochemical Characterisation. J. Cereal Sci..

[41] Greffeuille V., Mabille F., Rousset M., Oury F.-X., Abecassis J., Lullien-Pellerin V. (2007). Mechanical Properties of Outer Layers from Near-Isogenic Lines of Common Wheat Differing in Hardness. J. Cereal Sci..

[42] Chiremba C. (2012). Sorghum and Maize Grain Hardness: Their Measurement and Factors Influencing Hardness.

[43] Vega-Rojas L.J., Contreras-Padilla M., Rincon-Londoño N., del Real López A., Lima-Garcia R.M., Palacios-Rojas N., Rodriguez-Garcia M.E. (2016). The Effect of Maize Grain Size on the Physicochemical Properties of Isolated Starch, Crude Maize Flour and Nixtamalized Maize Flours. Agric. Sci..

[44] Yenge G.B., Kad V.P., Nalawade S.M. (2018). Physical Properties of Maize (*Zea mays* L.) Grain. J. Krishi Vigyan.

[45] Bhise S.R., Kaur A., Manikantan M.R. (2014). Moisture Dependent Physical Properties of Maize (*PMH-1*). Acta Aliment..

[46] Ashwin Kumar B., Rao P.V.K.J., Edukondalu L. (2017). Physical Properties of Maize Grains. Int. J. Agric. Sci..

[47] Seifi M.R., Alimardani R. (2010). The Moisture Content Effect on Some Physical and Mechanical Properties of Corn (Sc 704). J. Agric. Sci..

[48] Tarighi J., Mahmoudi A., Alavi N. (2011). Some Mechanical and Physical Properties of Corn Seed (Var. DCC 370). Afr. J. Agric. Res..

[49] Atere A.O., Olalusi A.P., Olukunle O.J. (2016). Physical Properties of Some Maize Varieties. J. Multidiscip. Eng. Sci. Technol..

[50] Ozturk T., Esen B. (2013). Physical and Mechanical Properties of Some Hybrid Corn Varieties. Int. J. Agric. Biol. Eng..

[51] Babic L.J., Radojcin M., Pavkov I., Babic M., Turan J., Zoranovic M., Stanisic S. (2013). Physical Properties and Compression Loading Behaviour of Corn Seed. Int. Agrophysics.

[52] Bolaji O.T., Awonorin S.O., Sanni L.O., Shittu T.A. (2018). Modelling of Mechanical Properties of Five Maize Varieties at Critical Processing Conditions in the Production of Fermented Slurry-Ogi. Int. J. Food Prop..

[53] AbdEl Maksoud M.A.F. (2009). Mechanical Properties of Corn Kernels. Misr J. Agric. Eng..

[54] Soyoye B.O., Ademosun O.C., Agbetoye L.A.S. (2018). Determination of Some Physical and Mechanical Properties of Soybean and Maize in Relation to Planter Design. Agric. Eng. Int. CIGR J..

[55] Zhang K., He Y., Zhang H., Li H. Research on Mechanical Properties of Corn Stalk. Proceedings of the AIP Conference.

[56] Kim T.H. (2000). Physical Changes in Maize (Zea mays L.) Grains during Postharvest Drying.

[57] Seifi M.R., Alimardani R. (2010). Comparison of Moisture-Dependent Physical and Mechanical Properties of Two Varieties of Corn (Sc 704 and Dc 370). Aust. J. Agric. Eng..

[58] Tavares L.M., Cavalcanti P.P., de Carvalho R.M., da Silveira M.W., Bianchi M., Otaviano M. (2018). Fracture Probability and Fragment Size Distribution of Fired Iron Ore Pellets by Impact. Powder Technol..

[59] Cavalcanti P.P., Tavares L.M. (2018). Statistical Analysis of Fracture Characteristics of Industrial Iron Ore Pellets. Powder Technol..

[60] Sun H., Zeng Y., Ye Y., Chen X., Zeng T. (2020). Abnormal Size Effect of Particle Breakage Probability under Repeated Impacts. Powder Technol..

[61] Maaß S., Kraume M. (2012). Determination of Breakage Rates Using Single Drop Experiments. Chem. Eng. Sci..

[62] Bwalya M.M., Chimwani N. (2020). Development of a More Descriptive Particle Breakage Probability Model. Minerals.

[63] Niedziółka I., Szymanek M. (2003). Utilization of Maize Grain for Industrial and Energetistics Purposes. Mot. Energy Rol.

[64] Gongora I.G., Dunoyer A.T., Garcia-Zapateiro L.A. (2018). Physical, Chemical and Biological Properties of Maize Variety Fr-28. Contemp. Eng. Sci..

[65] The Polish Committee for Standardization (Polski Komitet Normalizacyjny—PKN) (2012). Ziarno Zbóż i Przetwory Zbożowe—Pobieranie próbek PN-EN ISO 24333 (Cereals and Cereal Products—Sampling (ISO 24333:20010).

[66] Ștefan E.-M., Voicu G., Constantin G.-A., Stoica D. (2013). Variation of Crushing Characteristics by Compression of Wheat Seeds. Metal. Int..

[67] Nielsen S.S., Nielsen S.S. (2010). Determination of Moisture Content. Food Analysis Laboratory Manual.

[68] Pabst W., Gregorova E. (2007). Characterization of Particles and Partcile Systems.

[69] Markowski M., Majewska K., Kwiatkowski D., Malkowski M., Burdylo G. (2010). Selected Geometric and Mechanical Properties of Barley (*Hordeum Vulgare* L.) Grain. Int. J. Food Prop..

[70] Gudaczewski W., Konopka I., Kozirok W., Tańska M. (2012). Produkcja i Przechowalnictwo. Ziarna Zbóż i Nasion Oleistych.

[71] Gąsiorowski H. (2007). Kukurydza. Część 1. Właściwości Fizyczne. Przegląd Zbożowo-Młyn..

[72] Kruszelnicka W., Marczuk A., Kasner R., Bałdowska-Witos P., Piotrowska K., Flizikowski J., Tomporowski A. (2020). Mechanical and Processing Properties of Rice Grains. Sustainability.

[73] American Society of Agricultural and Biological Engineers (2008). ASAE S368.4 DEC2000 (R2008): Compression Test of Food Materials of Convex Shape.

[74] Chutkowski M., Zapała W. (2015). Zastosowanie Metody DEM Do Badania Wpływu Kształtu Ziaren Na Charakter Wysypu Materiału Rozdrobnionego Podczas Opróżniania Silosu. Inż. Apar. Chem..

[75] Sarker M.S.H., Hasan S.M.K., Ibrahim M.N., Aziz N.A., Punan M.S. (2017). Mechanical Property and Quality Aspects of Rice Dried in Industrial Dryers. J. Food Sci. Technol..

[76] Tavares L.M., de Almeida R.F. (2020). Breakage of Green Iron Ore Pellets. Powder Technol..

[77] Salman A.D., Ghadiri M., Hounslow M. (2007). Particle Breakage.

[78] Baumgardt S. (1975). On the Comparison of Results in Single Grain Crushing under Different Kinds of Load.

[79] Bochat A., Zastempowski M. (2019). Impact of the beater shredder design on the granulometric composition of the shredded grain material. Przemysl Chem..

[80] Bochat A., Zastempowski M., Kalaczynski T., Zoltowski M. (2018). Modelling of the grain materials “shredding process for the purposes of the beater shredders” designing. Proceedings of the 17th International Conference Diagnostics of Machines and Vehicles.

[81] Boikov A.V., Savelev R.V., Payor V.A. (2018). DEM Calibration Approach: Design of Experiment. J. Phys. Conf. Ser..

[82] Hlosta J., Jezerská L., Rozbroj J., Žurovec D., Nečas J., Zegzulka J. (2020). DEM Investigation of the Influence of Particulate Properties and Operating Conditions on the Mixing Process in Rotary Drums: Part 1—Determination of the DEM Parameters and Calibration Process. Processes.

[83] Pachón-Morales J., Do H., Colin J., Puel F., Perré P., Schott D. (2019). DEM Modelling for Flow of Cohesive Lignocellulosic Biomass Powders: Model Calibration Using Bulk Tests. Adv. Powder Technol..

[84] Voicu G., Biris S.-S., Stefan E.-M., Constantin G.-A., Ungureanu N. (2013). Grinding Characteristics of Wheat in Industrial Mills. Food Ind..

[85] Afkari-Sayyah A.H., Minaee S. (2004). Behavior of Wheat Kernels under Quasi-Static Loading and Its Relation to Grain Hardness. J Agric. Sci. Technol..

[86] Voicu G., Moiceanu G., Chitoiu M., Cardei P. Some Statistical Parameters for Miscanthus Giganteus and Salix Viminalis Grinding Using Hammer Mills. Proceedings of the Engineering for Rural Development.

[87] Tavares L.M. (2004). Optimum Routes for Particle Breakage by Impact. Powder Technol..

